# The Druze: A Population Genetic Refugium of the Near East

**DOI:** 10.1371/journal.pone.0002105

**Published:** 2008-05-07

**Authors:** Liran I. Shlush, Doron M. Behar, Guennady Yudkovsky, Alan Templeton, Yarin Hadid, Fuad Basis, Michael Hammer, Shalev Itzkovitz, Karl Skorecki

**Affiliations:** 1 Ruth and Bruce Rappaport Faculty of Medicine and Research Institute, Technion – Israel Institute of Technology, Haifa, Israel; 2 Laboratory of Molecular Medicine, Rambam Health Care Campus, Haifa, Israel; 3 Faculties of Biology and Engineering, Washington University, St. Louis, Missouri, United States of America; 4 Department of Emergency Medicine, Rambam Health Care Campus, Haifa, Israel; 5 Division of Biotechnology, University of Arizona, Tucson, Arizona, United States of America; 6 Department of Computer Science and Applied Mathematics, Weizmann Institute of Science, Rehovot, Israel; University of Canterbury, New Zealand

## Abstract

**Background:**

Phylogenetic mitochondrial DNA haplogroups are highly partitioned across global geographic regions. A unique exception is the X haplogroup, which has a widespread global distribution without major regions of distinct localization.

**Principal Findings:**

We have examined mitochondrial DNA sequence variation together with Y-chromosome-based haplogroup structure among the Druze, a religious minority with a unique socio-demographic history residing in the Near East. We observed a striking overall pattern of heterogeneous parental origins, consistent with Druze oral tradition, together with both a high frequency and a high diversity of the mitochondrial DNA (mtDNA) X haplogroup within a confined regional subpopulation. Furthermore demographic modeling indicated low migration rates with nearby populations.

**Conclusions:**

These findings were enabled through the use of a paternal kindred based sampling approach, and suggest that the Galilee Druze represent a population isolate, and that the combination of a high frequency and diversity of the mtDNA X haplogroup signifies a phylogenetic refugium, providing a sample snapshot of the genetic landscape of the Near East prior to the modern age.

## Introduction

Phylogenetic clustering of mtDNA haplogroups has been found to correlate with geography, such that different haplogroups often correspond to specific geographic origins [1]. For example, the L haplogroup is a hallmark of the African continent, where almost 95% of the inhabitants belong to the different lineages of this mtDNA haplogroup. Haplogroup L can also be found at low frequencies in other regions of the world due to migration events. Similarly, haplogroups A and B are predominantly found among Native Americans[1]. Haplogroup X is one of the exceptions to this pattern of limited geographical distribution, and is found at low frequencies among West Eurasians[2], northern groups of Native Americans[3], as well as in northern Africa and the Near East[4]. A very high global genetic diversity has been reported for haplogroup X[4].

Haplogroup X is further divided into two subclades. Subhaplogroup X1 was found to be largely restricted to the Afro-Asiatic-speaking populations of northern Africa and the neighboring areas, suggesting a possible geographic diffusion of X1 along the coast of the Mediterranean and the Red Sea. Subhaplogroup X2 is characterized by a much wider geographic range, but at the same time by very low frequencies in the populations of the regions where it is found[4]. No population or geographic region has been identified to date, in which haplogroup X and its major subhaplogroups are found at both high frequency and high diversity, which could provide a potential clue as to their geographic origin. Here we suggest that the Druze population of northern Israel may represent just such a population.

The Druze population has a unique historical, social and demographic structure, which is closely connected with their religion. The contemporary Druze population constitutes a small minority in four countries of the Near East: Syria, Lebanon, Israel and Jordan. In total, the estimated population number is fewer than 1,000,000 in the Near East and fewer than 100,000 in the Druze Diaspora. The Israeli Druze population is estimated at 150,000, and is distributed over three geographical subregions: the Carmel, the Galilee, and the Golan Heights. It has been postulated according to historical records that the origin of the Druze in each of these regions is different. Although the Druze represent a small percentage of the total population of the countries of the Near East in which they reside, their concentration in mountainous districts has produced a compact social structure, resulting in a nearly exclusive majority in some geographical regions, and therefore a low frequency of admixture with other populations. Druze customs strongly favor marriage within the same village or the same geographical area[5]. This social structure has turned the Druze into transnational isolates – a population which remains genetically isolated largely through the social practice of endogamy and consanguinity, despite being found in the midst of larger population majorities in multiple nationalities or countries[6]. Furthermore, unlike other monotheistic religions, the Druze tenets strictly close their religion to new adherents, thus forbidding admixture with other populations.

Previous studies described a high frequency with a low diversity of both the X1 and X2 subhaplogroups in the Druze population[4], [7]. This was attributed to a founder effect, genetic drift, and population expansion[4]. These explanations contradict Druze oral traditions, which claim that while the religion was revealed at the time of the “Dawa” (1017 A.C.E.), its adherents came from heterogeneous ancestral origins dating back further in antiquity. We sought to resolve this apparent contradiction, and at the same time re-examine the overall mtDNA and Y-chromosome diversity using a sampling strategy which might be more appropriate for the social structure and marriage practices of the Druze population. In so doing, we uncovered an unexpectedly high diversity of Druze X-haplogroup lineages, which together with its high frequency suggest that this population provides a glimpse into the past genetic landscape of the Near East, at a time when the X haplogroup was more prevalent.

## Results

### Population Sample

Altogether we sampled 311 different paternal households from 20 Druze villages in Northern Israel, (Table S1 [5], [8]), and 208 surnames were identified, consistent with the fact that certain kindreds have adopted the same surname, despite family records clearly indicating distinct paternal origins. We adopted the surname and household directed sampling strategy in order to avoid multiple sampling from the same kindred, which would reduce the ability to identify the maximum range of different parental lineages (see below and Figure S1). Sampling was carried out according to paternal rather than maternal households, since the Druze kindred structure is based on paternal family identity, though knowledge of maternal ancestry is often also retained. Moreover, a high degree of consanguinity (47% first cousin marriages[9]) and local endogamy, generate a situation in which each paternal household in a specific geographic region is a subpopulation isolate in which there is a strong correlation between paternal and maternal household identity[10]. Thus in the absence of a readily available approach to ascertain distinct maternal households, a paternal household based sampling strategy also facilitates identification of the maximum number of diverse maternal lineages in a given sample set.

### Population Genetic Indices

In 311 samples, comprising 208 surnames. 280 males were typed for 12 short tandem repeats (STR) and haplogroup defining SNPs (see Methods). We found 105 Y-chromosome haplotypes as defined by the 12 STRs (see Methods ); calculated genetic diversity [11] H^^^ = 0.979±0.002).

Analysis of Nei's genetic diversity (H^^^) among the different Druze subregions for both NRY lineages (for definition see Methods) and mtDNA lineages (defined by nucleotides 16024–16569 and 1–310), revealed similar diversities for both the NRY and the mtDNA for all Druze geographic subregions (Table S5), excluding matrilocality or patrilocality among the Druze[12]. Analysis of NRY haplogroup frequencies demonstrated that the most frequent NRY haplogroup among the Druze was J (33.2%) followed by haplogroup E (18.9%) and haplogroup R (18.2%) (Table S6). Analysis of population substructure by NRY haplogroups among different geographic regions indicated a significant difference in the frequency of haplogroup K among the different localities. Haplogroup K frequency in the Galilee region was 11% (21/183) in comparison to 0 (0/33) in the Carmel region (p = 3.3*10^−9^). There were 10 STR haplotypes and 7 lineages among the 24 haplogroup K individuals (H^^^ = 0.71). In contrast to the overall high NRY haplogroup diversity for the entire sample, village based pockets of low diversity were observed, consistent with local paternal founder effects. For example, the village of Beit -Jaan included 5 samples from haplogroup K, all of which belonged to the same lineage (H^^^ = 0). Similar phenomena were observed in the village Sajur, in which all 5 haplogroup K samples belonged to the same lineage (H^^^ = 0). This pattern of overall high diversity with local regions of low diversities was also evident for other villages and other NRY haplogroups, as well mtDNA haplogroups (data not shown).

Mitochondrial DNA analysis (nucleotides 16024–16569 and 1–310) carried out on all samples, revealed 106 haplotypes (H^^^ = 0.979±0.003) (Table S2 and S3). In their previous Israeli Druze database of 45 randomly sampled individuals, Macaulay et al.[7] found 25 haplotypes, based on part of the HVS-I region (nucleotides 16090–16365), yielding a calculated genetic diversity of H^^^ = 0.94±0.02. Analysis of the same HVS-I region in our Druze database revealed 82 haplotypes with an estimated genetic diversity H^^^ = 0.968±0.004 (*P*<0.005 for comparison of genetic diversity in the two sample sets). It is clear that the calculated genetic diversity for a given genomic region will depend upon the sampling approach and sample size. A small sample set which might include individuals from the same kindred or from a confined geographic region in which there is a high rate of consanguinity and endogamy is expected to yield a lower calculated genetic diversity than a larger sample set gathered from individuals belonging to different parental households from different subpopulations.

In order to exclude the effect of sample size alone in generating this difference, we conducted a bootstrap analysis designed to account for the different sample sizes in the two studies (see Methods), and found that, the difference in diversity remained significant (P<0.04). We used a simplified analytical model to assess the probability of obtaining a diversity level with random sampling, comparable to that obtained by surname directed sampling (see Methods). Applying random sampling to a set of 45 samples in a population comparable haplotypes frequencies as observed in the Druze, yields 27.3±2.6 haplotypes, similar to the 25 haplotypes observed by Macaulay et.al [7], supporting the fidelity of this numerical model. Using this model, we found that the probability of obtaining maximum gene diversity under a random sampling scheme is extremely small compared to directed sampling (P<0.0001).

**Figure 1:**
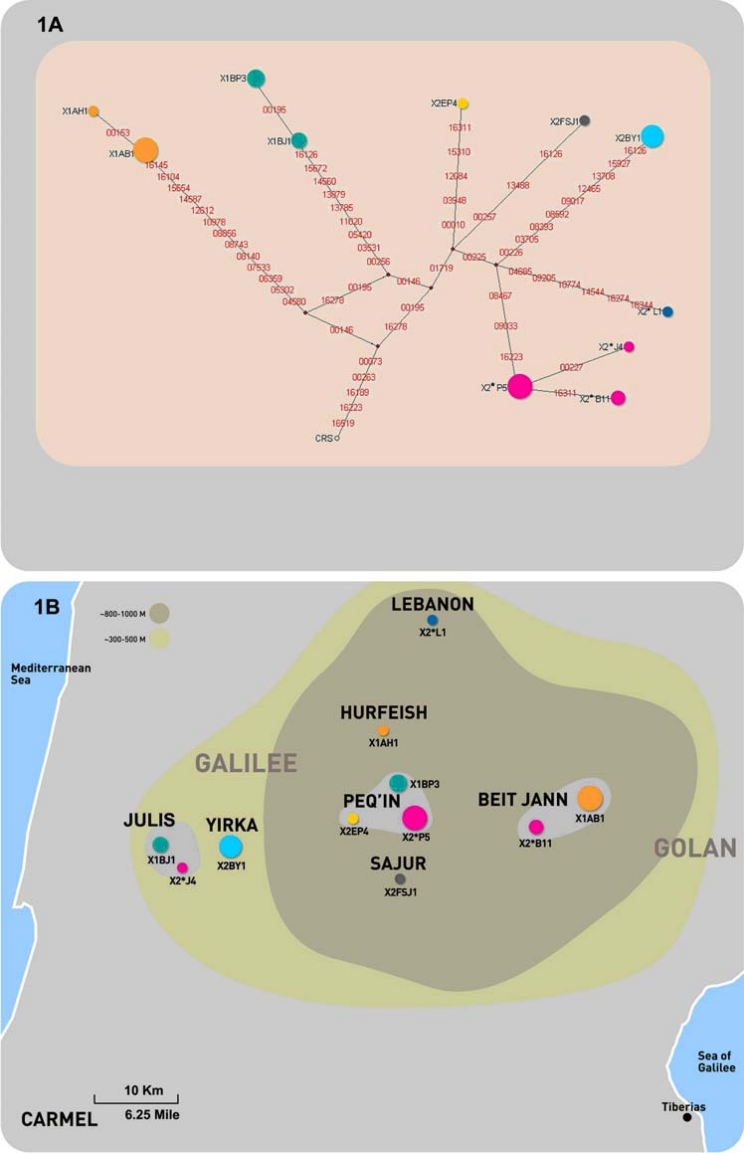
Haplogroup X in Galilee Druze. Figure 1A: Median-joining (MJ) network of the 41× haplogroup samples. The MJ[51] algorithm was implemented within the Network 4112 program. Areas of the colored nodes are proportional to haplotype frequencies. Each different color represents a different lineage. Nomenclature of node name: the first three characters of each node indicate the X haplogroup haplotype nomenclature according to Reidla et.al[4]. Asterisks designate samples whose genotype does not match any of the currently designated subhaplogroups. The fourth character defines the Druze Galilee village in which the haplotype was the most prevalent, according to maternal ancestry. B = Beit Jaan; H = Hurfeish; J = Julis; L = Lebanon; P = Peq'in; S = Sajur; Y = Yirka. The fifth character indicates the haplotype serial number. CRS = the Cambridge Reference Sequence. Nucleotide changes are specified by suffixes only for transversions; insertions and deletions are not designated. Figure 1B: The geographic distribution of the various Druze X haplogroup lineages in the Galilee region. Node names and colors are identical to those in figure 1A.

The effect of sampling strategy was particularly evident for the mtDNA X-haplogroup, in which we found both a high frequency (13.1%, 41/311 samples) together with a high diversity of haplotypes, as defined in Macaulay et al(7) on the basis of sequence variation at the HVS-I region (nucleotides 16090–16365) (Table 1). Macaulay et al[7] also found a high frequency of the X haplogroup (26%, 12/45 samples) in the Druze, but with a significantly lower diversity (p<0.00005) for both X1 and X2, (p value was calculated using t-test combined with bootstrap standard error analysis according to Nei^16^) , and proposed a bottleneck or founder effect. Given the different sampling strategies, we sought to determine the total number of different mtDNA X haplogroup lineages in our sample set. In order to determine the total number of distinct lineages, we followed the approach previously described for several other populations[13]–[15], of first sequencing the complete HVS-I and HVS-II D-loop sequences for all samples in the haplogroup, followed by complete sequencing of the entire mtDNA for each of the D-loop defined haplotypes. We found that the 41 Druze X-haplogroup sequences were comprised of 11 different D-loop defined haplotypes. Accordingly, we conducted full mtDNA sequencing for each of these 11 different D-loop defined haplotypes and found a total of 7 different coding region defined lineages, 2 of which belonged to major subhaplogroup X1 and 5 of which belonged to major subhaplogroup X2 (Figure 1a).

**Table 1:** Haplogroup X Frequency and Genetic Diversity in Druze samples of the Current Study, Compared with Macaulay et al[7].

	X1^a^	X2^a^	X1^b^	X2^b^
Overall Frequency	17/311 (5.4%)	24/311 (7.7%)	7/45 (15.6%)	5/45 (11.1%)
Galilee Region Frequency	17/182 (9.3%)	24/182 (13.2%)	N\A	N\A
Genetic diversity^c^	0.4412 +/−0.0977	0.7029 +/−0.0623	0	0.40+/−0.03
Genetic diversity^d^	0.5221 +/−0.1009	0.7049 +/−0.062	N\A	N\A

a = current study; b = Macaulay database; c = genetic diversity[11] according to HVS-I 16090–16365; d = genetic diversity according to full mitochondrial sequence.

Allele frequency approaches are also often used to ascertain population bottlenecks and expansions[16]–[19]. Since the application of such estimates to a non-random sample has not been ascertained, we have restricted ourselves to analysis of the mean mismatch distribution for the HVS-I segment (16090–16365)[18], [20]. The mean number of pairwise differences[21] in the sample set was 5.42 +/−2.56 (n = 311), and the distribution of the pairwise difference was unimodal as expected in populations that have undergone population expansion[18]. However since there was no excess of zero or one pairwise nucleotide differences, and since the estimated time of expansion Tau = 5.94 (∼60,000 years ago) did not differ from other Near East populations[16], the probability of a recent founder event and bottleneck is small.

### Haplogroup X and the Druze Refugium

The finding of a surprisingly high frequency and high diversity of X haplogroup lineages, and the rejection of a recent bottleneck or founder effect, encouraged us to conduct a more detailed analysis of the distribution of the X haplogroup lineages according to geographic sub-regions. Using a hypergeometric test[22] significant enrichment of haplogroup X was localized in the Galilee region of the Israeli Druze population (Figure 1b). We found that 39 of 41 haplogroup X Druze individuals were from the Galilee heights (Table S2), corresponding to 21.4% (39/182) of the samples from that region. Enrichment analysis[22] revealed that both X1 and X2 were highly enriched in this region (*P* = 9.3*10^^−5^ and  = 3*10^^−4^, respectively). This finding remained significant after Bonferroni correction for multiple hypothesis testing. In another independent unpublished large Near East sample set, which also included 77 Israeli Druze samples from the Carmel region, only one sample belonged to the X haplogroup (Behar DM et. al, submitted). One particularly exceptional example of both high diversity and high frequency of haplogroup X was the Galilee heights village of Peq'in, where 6 of 17 households (35.3%) belonged to four distinct lineages of haplogroup X (X1a, X1b, X2e and X2*), yielding a calculated genetic diversity for both X1 and X2 of 0.667±0.02. This village, is believed to be one of the oldest Druze villages in Israel, and is mentioned in historical records dating from the 13th century. These records specifically note that certain villages in the Galilee accepted the Druze religion during the “Dawa” and never left this geographic region[23], [24].

An attractive hypothesis to explain this geographic concentration of lineages which diverged remotely within an ancient haplogroup (and which are not identified in other populations of the region) is that these Galilee Druze individuals represent the refugium of an ancestral group with high diversity and high frequency of haplogroup X, which was more prevalent in the region in antiquity, and from which the global diversity of X mtDNA haplogroup emerged. Alternative hypotheses could be non-random sampling effects during the original establishment of the Druze population. We now consider these alternatives.

First consider the hypothesis that the current population genetic structure is the result of a random or non-random migration process contributing to original members of the Druze religion. Indeed, Druze tradition considers the population to have been constituted from many diverse source populations. Various studies have shown that when a new colony is formed from several potential source populations, either lower[25] or higher[26] genetic differentiation can occur depending upon how colonizing groups of individuals are formed, and depending upon the quantitative relationship between colonization and migration. One possible model for the colonization of a new or vacant habitat occurs when some subpopulations in nearby geographic regions contribute migrants to a common pool, the “migrant pool,” from which colonists are drawn at random to fill vacant sites, with mixing of individuals from different populations[27]. Historical records are unclear as to the identity of the original Druze adherents (the original subpopulations which donated to the migrant pool). Accordingly, we compared the sum square distance of the haplogroup frequency profile of the Druze in our study, to those reported for other nearby Near East populations[28]. The populations with the smallest genetic distances to the Druze were: Turks, Armenians, Iranians and Egyptians. Historical records suggest a strong Druze connection with Turks and Egyptians during the “Dawa” period. Therefore, we tested the possibility of obtaining an X haplogroup frequency as high as observed in our study (41/311), from random sampling of Turkish individuals as a metapopulation contributing to the migrant pool, (Turks have a current X haplogroup frequency of 6/218[28]). The distribution of X haplogroup individuals in such a sampling scheme follows a hypergeometric distribution and proved to be very unlikely (p<10^−5^), using a wide range of both source and sampled populations. The probability for the migrant pool model was even lower when calculated for Egyptians, Armenians and for different combinations of these postulated source populations for such a colonization process. Furthermore, comparison of the Druze X haplogroup sequences to Turkish and Egyptian X haplogroup sequences[4] revealed different lineages whose coalescence antedates the “Dawa” period.

**Figure 2:**
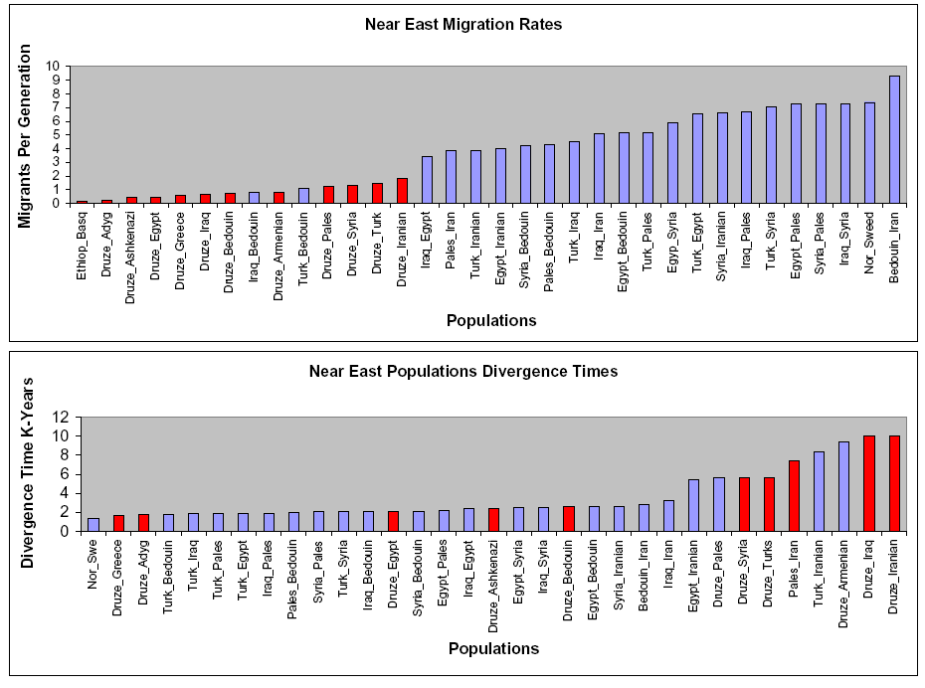
Migration Rates and Population Divergence Times among Near East Populations. Demographic modeling using the IM application[40] applied to mtDNA HVS-I sequences (nucleotides 16067–16384) from various Near East populations. Red bars = Druze with other Near East populations. Purple bars = Near East populations among themselves. Migration rates represent the mode of the posterior distribution of the number of migrants per generation. The divergence time is in thousand of years. The following populations were used: 311 Druze from the current study. Sequences reported in Macaulay et al [7]: Egyptians (Egypt) 67 samples; Iraqis (Iraq) 116 samples; Syrians (Syria) 69 samples; Palestinians (Pales) 110 samples; Turks (Turk) 218 samples; Armenians (Armenian) 191 samples; Adygei (Adyg) 50 samples; Greeks (Greece) 65 samples; Swedes (Swe) 32 samples; Nowegians (Nor) 231 samples; Basques (Basq) 156 samples. Sequences reported by Hammer et.al [53]: Bedouins (Bedouin) 58 samples. Sequences reported by Metspalu et.al [54]: Iranian (Iran) 436 samples. Sequences reported by Thomas et.al [55]:Ashkenazi Jews (Ashkenazi) 78 samples Ethiopians (Ethiop) 74 samples.

To further exclude the possibility of migration as a dominant force shaping the genetic landscape of the Druze, we have applied a demographic modeling strategy. We have used the isolation with migration model [29], which is a Markov chain Monte Carlo calculation using DNA sequence data to estimate the relative effects of migration and isolation on genetic diversity in a pair of populations. Using this model, we demonstrate that the Druze have low migration rates with all nearby populations. The highest migration rates of Druze with other populations were observed with Iranians and Turks, but still these migration rates were considerably lower in comparison to migration rates of other Near East populations among themselves (Figure 2). With regard to the time of population divergence, the Druze were most closely related to Greeks, Adygei, Egyptians and Ashkenazi Jews (AJ). Other maximum likelihood estimates of migration rates and dates of population divergence, inferred from the mtDNA data, are presented in Figure 2. Of interest, even within the Druze, analysis of maternal migration rates revealed, low migration values for Galilee Druze with other Druze regions, with the exception of Lebanon (Figure S2).

We also used a numerical model to test and exclude the possibility of non-random migration and colonization [30]–[32]such as might result from fission of kin structured and hence correlated samples from nearby populations. The probability values for non-random migration and colonization were all <0.05 for a wide range of population parameters (Table S4). Therefore it seems highly unlikely that the contemporary Galilee Druze haplogroup X subpopulation reflects either random migration as in the island migration model[33] or a colonization process originating from nearby Near-Eastern populations.

A clear expectation for a population refugium as suggested by the Druze mtDNA haplogroup X analysis is the finding of high diversity also among other haplogroups that were more prevalent in the past and which have survived in the population refugium. Also, this high diversity specific to the refugium population, should not be shared with nearby populations which are not part of the refugium. Therefore we extended the mtDNA analysis to other Druze haplogroups. Complete mtDNA sequencing for each of the D-loop defined haplotypes was carried out for haplogroups H and K samples, which together with haplogroup X were the 3 most prevalent haplogroups among the Druze population. For 32 D-loop defined haplotypes within haplogroup H, we observed 26 different coding region variant defined lineages, of which 12 represent novel lineages (Table 2). For 10 D-Loop defined haplotypes within haplogroup K, we observed 10 different coding region variant defined lineages, 5 of which novel K1a sub-lineages (Table 2).

**Table 2:** Haplotype diversity among Druze Haplogroup K and H as defined by D-Loop and Full mtDNA sequence.

Haplogroup	# of D-Loop Defined Haplotypes	# of Full mtDNA sequence Defined Haplotypes
H*	16	14
H1	4	3
H4b	5	3
H5	1	1
H7	5	4
H8	1	1
K1a*	5	5
K1a4b	1	1
K1a5	2	2
K1a6	1	1
K1b1	1	1

* = lineages that were not defined under the current known mtDNA tree topology.

## Discussion

Our findings of high diversity and high frequency of X haplogroup concentrated among the Galilee Druze provides new insights regarding both sampling methodologies in population genetics, and the understanding of the effects of sociological patterns on the population genetic landscape of the Near East.

The findings provide a formal demonstration that in an endogamous and consanguineous population, calculated mtDNA genetic diversity is significantly lower using random sampling than using paternal or maternal household directed sampling. Therefore, in order to obtain a comprehensive phylogenetic picture of lineage diversity in a sample set from the Druze population or populations with similar socio-demographic patterns, it is necessary to either collect a very large random sample, or to use a directed sampling strategy to ensure coverage of all of the regionally endogamous cryptic subpopulations. Random sampling taken from a regional and small sample within an endogamous subpopulation, will be relevant only for that specific subpopulation as might have been the case in the Macaulay et.al[7]sample, and cannot necessarily be extrapolated to the population as a whole. This is also reflected in the diversity parameters, which we have obtained using the directed sampling approach, and which do not support a recent population bottleneck or founder effect for the Druze population as a whole. It should be noted though, that directed sampling might miss localized founder effects and bottlenecks, since it does not provide the real haplotype frequencies, but rather is designed to uncover as many diverse lineages as possible for a given sample size. The decision of which sampling method is more appropriate depends on the scientific question being addressed. With regard to population substructure and genetic distances directed sampling is more appropriate to uncover cryptic diversity and regional substructure in populations such as the Druze, characterized by high levels of endogamy and local founder effects. In studies designed to uncover population allele frequencies, or population based association studies with complex clinical phenotypes, the first step will have to be elucidation of the population substructure by directed sampling, followed by random sampling from a regional subpopulation.

The absence of a recent demographic bottleneck or population founder effect for the Druze population is consistent with their oral tradition of heterogeneous ancestral origins, and also is consistent with the pattern of Mendelian genetic disease in the population. Rather than finding an excess of genetic disease with monogenic inheritance distributed throughout the population, rare monogenic recessive diseases are kindred or community specific and causative mutations have been successfully mapped in many cases using homozygosity mapping strategies[34], [35]. This contrasts with the pattern observed in populations with recent bottlenecks, such a Ashkenazi Jews, in whom founder mutations have been mapped, which are distributed throughout all communities of the population[36]–[38].

Lineage analysis within the mtDNA X-haplogroup was particularly enlightening. It should be noted that the estimated coalescence times for the major mtDNA X subhaplogroups X1 and X2 are 42,900±18,100 and 17,900±2,900 respectively[4]. It is striking that those different lineages (from the same parental haplogroup) whose genetic divergence date back more than ten thousand years would remain so concentrated within such a small geographic region. Mutation rates for the mtDNA coding region[39], are not consistent with the possibility that this number of different coding region defined lineages within haplogroup X could have resulted from the recent expansion of a monophyletic clade within the past 1000 years. Rather this combination among the Druze, of a large number of lineages, together with a high frequency of the haplogroup in which these lineages are found, suggests descent from an ancestral population, in which the X haplogroup was more abundant than it is in the contemporary Near East, and which reflects the prevailing Near East genetic landscape at that time, antedating the establishment of the Druze religion in 1017 A.C.E. This supports the notion that the Druze represent a refugium of the population genetic structure from the time period prior to the “Dawa”, and also confirms the hypothesis of high endogamy among the Druze. The refugium hypothesis based on mtDNA haplogroup X analysis was corroborated by the finding of high diversity for the Druze mtDNA haplogroups H and K, with the added finding of novel lineages not shared with nearby populations.

Furthermore, the formal rejection of the alternate hypothesis relating to immigration to the region of individuals sharing the same mtDNA haplogroup but with lineages that diverged in antiquity, further strengthens the Druze refugium model. Although, we cannot exclude the possibility that some ratio between non-random migration and colonization did occur during and following the “Dawa” period and generated the differences in haplogroup frequencies among the current Druze subregions, and between the Druze and other populations, this explanation is highly unlikely in face of the demographic modeling results. The overall low migration rate between the Druze and all other nearby populations (Figure 2) cannot explain the high diversity and high frequency of X haplogroup lineages in the Galilee region. Low migration rates were also evident between the Galilee Druze and Druze from other subregions. The finding of the enrichment of the NRY haplogroup K among the Galilee Druze with no detection in samples from other subregions, further supports the relative isolation of this region, even among the Druze. Taken together these findings support the hypothesis that the Galilee Druze are a further more isolated subpopulation of the Druze, who in turn represents a refugium of the population genetic architecture of the Near East in antiquity.

Demographic modeling can also provide estimates of divergence times for populations with shared ancestries. The demographic modeling in the current study indicates most recent divergence of the Druze from an ancestral population shared with Egyptians, Ashkenazi Jews, Adygeis and Greeks (Figure 2). The Egyptian shared ancestry is also consistent with Druze oral tradition. The migration rates of the Druze with these populations are exceedingly low, and this can be attributed to endogamy and geographic isolation following divergence. It should be kept in mind however, that the computational algorithms used for the demographic modeling are designed for models involving an ancestral population which split and maintained constant migration rate among the two daughter populations. Such a simplified model does not take into account the effect of shared party migration, and therefore would tend to overestimate the migration rate, and underestimate the divergence time[40]. Therefore the Druze would seem to have an even greater degree of genetic isolation, than indicated by these results of the demographic modeling.

The historical events and time frame for the loss or dilution of haplogroup X individuals is consistent with the population upheavals and patterns of migration that have characterized the Near East during the past two millennia at least [41], [42]. The preservation of this refugium of mtDNA lineage diversity among the Druze, mainly due to genetic isolation may be the result of their location in relatively more defensible mountainous regions, and the practice of conciliation with governing authorities and dissimulation called the “Taqiyya”[43], or due to other factors which facilitated preservation of societal integrity during periods of demographic and political change in the region.

### Conclusions

Our findings suggest that the Near East maternal genetic landscape differed substantially in the past from its current structure, and was enriched in diverse lineages of the mtDNA X haplogroup. These findings have been uncovered due to the unique demographic features of the Druze population, and the adjusted sampling method employed in the current study. The combination of a high frequency and diversity of the Druze mtDNA haplogroup X lineages, in a confined geographic region, and the low migration rate with nearby populations make it unlikely that this diversity was imported. It is thus likely that the global diversity of this haplogroup evolved in the Near East and adjacent regions of western Eurasia, during a long incubation period coinciding with and following the most recent out of Africa expansion as dated by mtDNA coalescence simulations[44]. The Druze population of the Galilee represents a contemporary refugium of this past genetic landscape.

## Materials and Methods

### Population Samples

We employed a paternal household-based sampling method (Figure S1) from 20 Druze villages in northern Israel, (Table S1). Individuals with different surnames from the same village were sampled. In several cases, individuals from the same village having the same surname were sampled if they could show that their households were unrelated, despite sharing an identical surname, thus yielding a total of 311 samples for analysis. The 41 mtDNA X haplogroup samples were available for complete mtDNA sequencing, of which 11 could be assigned to different HVS-I and HVS-II D-loop sequence based haplotypes and were therefore subject to complete mtDNA sequence. Samples sharing the same HVS-I and HVS-II D-loop sequence as noted above were assigned to the same mtDNA X-haplogroup lineage. DNA samples were extracted from buccal swabs, with written informed consent according to procedures approved by the Institutional Human Subjects Review Committee. Each subject reported the birthplace of his/her maternal and paternal ancestry.

### Genotyping

Sequences were determined using the ABI Prism Dye Terminator cycle-sequencing protocols developed by Applied Biosystems (Perkin-Elmer), and according to previously reported protocols[45]. Sequence traces were analysed using SEQUENCHER software. Mutations were scored relative to the revised Cambridge Reference Sequence (rCRS)[46]. The novel haplogroup X complete mtDNA sequences reported are available in GenBank (accession numbers EU600318-EU600370).

We followed the recommendations of Hammer and Zegura 2002[47] in typing NRY binary markers that define all 18 major haplogroups on the YCC (2002) tree and their sub-branches. The genotypes for these sites were determined by allele specific PCRs. PCR protocols for detection of these polymorphisms have been previously reported by Karafet et al.[48]. For the microsatellite analysis, twelve short tandem repeats (Y-STRs: *DYS19, DYS388, DYS389I, DYS389II, DYS390, DYS391, DYS392, DYS393, DYS426*, *DYS439, DYS438 and DYS457*) were genotyped in two multiplex reactions following the protocol of Redd et al. [49]. PCR products were electrophoresed on a 3730XL Genetic Analyzer (Applied Biosystems), and fragment lengths were converted to repeat number by the use of allelic ladders. The data were analyzed with Genescan (v. 3.7, Applied Biosystems) and Genotyper (v. 1.1, Applied Biosystems). We define *DYS389CD* as equivalent to *DYS389I*, and we define *DYS389AB* as equivalent to *DYS389II* minus *DYS389I*.

### Nomenclature

We adopted the consensus haplogroup nomenclature schemes for both the NRY and mtDNA [50]. For mtDNA numbers 1–16569 refer to the nucleotide positions in the rCRS[46]. In the case of the NRY the term lineage is used to denote a cluster of related evolving STR-based haplotypes within a haplogroup. NRY lineages were defined for each haplogroup. The MJ[51] algorithm was implemented within the Network 4112 program to draw the MJ tree of each NRY haplogroup by using the 12 STR data. The next step in the lineage assignment included calculating coalescence time for each of the sub-branches using Network 4112 program. The average mutation rate for the 12 STRs was 3019 years per mutation[52]. Using this approach, lineages were defined as a group of STR defined haplotypes within a haplogroup that coalesce to more than 1000 years. The cutoff of 1000 years was used in order to estimate the number of lineages that were included in the Druze genetic pool before the “Dawa” period.

In the case of mtDNA, haplotypes and lineages can relate to either the control region or the complete mtDNA sequence data[15].

### Statistical analysis and population genetics indices

In order to evaluate the differences in mtDNA genetic diversity between the current dataset and that reported previously[28], we have used t-test combined with bootstrap standard error analysis according to Nei [11]. To assign a p-value to the null hypothesis that the low diversity of previous studies [4], [7] is a result of smaller sample size, we randomly chose 1000 sample sets of identical sizes (*n* = 45) from the current data set and calculated the resulting gene diversity. The p-value is the fraction of samples in which the gene diversity was lower than that in the previous study.

We used a simplified analytical model to assess the probability of obtaining a diversity level with random sampling, comparable to that obtained by surname directed sampling. For simplicity we use the number of haplotypes, rather than gene diversity, as a measure of diversity. We consider an ideal situation where surname households are completely correlated with mitochondrial lineages. Considering a population of N haplotypes (or mitochondrial lineages), each containing x individuals. We sample m individuals (without loss of generality m≥N). Given a non-random sample according to surname k = N haplotypes are expected for any sample set. The probability of obtaining k haplotypes using random sampling is:
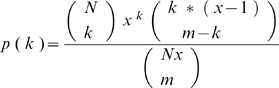
Using this formula, the p value – probability to obtain N haplotypes (maximum diversity) under a random sampling scheme can be calculated. We also used a more detailed model in which we included the relative weights of each one of the observed haplotypes, so the number of haplotypes in each lineage is not constant. We simulated sampling (m) individuals from this weighted distribution over haplotypes and numerically calculated the probability to obtain N haplotypes.

The Arlequin software package[21] was used to estimate mean pairwise differences and mismatch distribution parameters.

To assess the probability that the fraction of X haplogroups in the Druze population may arise from a sampling of a larger source population, we assumed a source population of size N, f_s_ of which are X haplogroup, and a target population (the original Druze) of size n, f_t_ of which are X haplogroup (for example f_s_ = 6/218 for the Turks and f_t_ = 41/311 for the Druze). The distribution of X haplogroup individuals in such a sampling scheme follows a hypergeomtric distribution, from which a p-value can be obtained. We estimated this for source populations of 10,000–10,000,000 and target population of size 100–100,000.

### Demographic Modeling

The simulation tool IM [40] was used to calculate maternal migration rates and population divergence times between pairs of populations. The mtDNA nucleotide sequence 16067–16384 was used for this analysis and applied to various populations (Figure 2). Run-time parameters included a maximum migration rate of 10, a maximum divergence time of 10 Ne generations, and a burn in of 100,000 steps followed by a run of at least 10,000,000 steps with a linear heating scheme of increment 0.1. The following standard settings were used (-q1 10 -m1 10 -m2 10 -t 10 -b 100000 -L 0.5 -s123 -p45). All pairwise population sample comparisons were replicated with different random number seeds. The mode of each marginal posterior distribution was considered as a point estimate of the corresponding parameter value.

## Supporting Information

Table S1 (0.05 MB DOC)

Table S2 (0.52 MB DOC)

Table S3 (0.04 MB DOC)

Table S4 (0.05 MB DOC)

Table S5 (0.04 MB DOC)

Table S6 (0.04 MB DOC)

Figure S1 (0.28 MB DOC)

Figure S2 (0.14 MB DOC)
