# Supplementary material for: The Druze: A Population Genetic Refugium of the Near East
Source: PLoS One. 2008 May 7;3(5):e2105. doi: 10.1371/journal.pone.0002105 (PMC2324201; doi:10.1371/journal.pone.0002105)
Supplement: Table S1 — (0.05 MB DOC) [file pone.0002105.s001.doc]

**Table S1**: Israeli Druze sample from different geographic regions

| **Village** | **Region** | **Sample Size** | **Population Size 1887 A.C** | **Population Size 2005** |
| --- | --- | --- | --- | --- |
| Abu Sinan | Galilee | 3 | 330 | 3900 |
| Beit Jaan | Galilee | 41 | 1215 (240) | 9600 |
| Beq’ata | Golan | 14 | Unknown | 5200 |
| Daliyat al-Karmel | Carmel | 23 | 744 | 20500 |
| Ein Qiniyye | Golan | 1 | Unknown | 1700 |
| Hurfeish | Galilee | 11 | Unknown | 4700 |
| Jat | Galilee | 9 | 105 (21) | 1491 |
| Jordan-zarka* | Syria | 1 | Unknown |  |
| Julis | Galilee | 12 | 360 | 5100 |
| Kisra | Galilee | 8 | 70 (14) | 1116 |
| Lebanon* | Lebanon | 29 | Unknown | 450000 |
| Majdal Shams | Golan | 20 | Unknown | 8400 |
| Mas’ade | Golan | 2 | Unknown | 3100 |
| Mughar | Galilee | 29 | 625 | 10400 |
| Peq’in | Galilee | 17 | 850 (170) | 3700 |
| Rame | Galilee | 11 | 425 | 2200 |
| Sajur | Galilee | 8 | 190 | 3400 |
| Shefar’am | Galilee | 7 | 440 | 4500 |
| Sumei | Galilee | 6 | 280 (50) | 4465 |
| Syria* | Syria | 26 | Unknown | 400000 |
| Isifya | Carmel | 12 | 480 | 6000 |
| Yanuh | Galilee | 7 | 245 | 3430 |
| Yirka | Galilee | 14 | 1285 | 11800 |
| Total |  | 311 | Unknown | 917611 |

* The samples from Jordan, Lebanon and Syria are immigrants from these countries who were sampled in Israel.
