# Supplementary material for: The Druze: A Population Genetic Refugium of the Near East
Source: PLoS One. 2008 May 7;3(5):e2105. doi: 10.1371/journal.pone.0002105 (PMC2324201; doi:10.1371/journal.pone.0002105)
Supplement: Table S2 — (0.52 MB DOC) [file pone.0002105.s002.doc]

**Table S2**: Druze Haplotypes and Haplogroups According to MtDNA and NRY.

| Serial # | Maternal Origion | Haplogrop Mt | HVS-I (16024-16383) | Middle (16384-00056) | HVS-II (00057-00310) | Haplogroup Y | Summary Y STR |
| --- | --- | --- | --- | --- | --- | --- | --- |
| 1 | Beit Jaan 1 | H | 172 355 | 400 | 263 | K-M9 | 11 12 13 15 14 31 14 23 10 13 9 14 |
| 2 | Beit Jaan 1 | H | CRS | 519 | 93 263 291.1 | R-P25 | 12 12 13 17 13 29 14 24 11 13 12 15 |
| 3 | Beit Jaan 1 | H | 187 218 | CRS | 263 | R-P25 | 12 12 13 16 13 29 14 24 11 13 12 15 |
| 4 | Beit Jaan 1 | H | CRS | 519 | 93 263 291.1 | R-P25 | 12 12 13 17 13 29 14 24 11 13 12 15 |
| 5 | Beit Jaan 1 | H | 93 | CRS | 153 195 263 | J-M172 | 11 14 11 15 13 31 15 26 10 12 9 15 |
| 6 | Beit Jaan 1 | H | 172 355 | 400 | 263 | R-P25 | 12 12 13 18 13 29 14 24 11 13 12 15 |
| 7 | Beit Jaan 1 | H | 240 | 519 | 146 263 | R-P25 | 12 12 13 16 13 29 14 24 11 13 12 15 |
| 8 | Beit Jaan 1 | H | 93 183D | CRS | 153 195 263 | R-P25 | 12 12 13 16 13 29 14 24 11 13 12 15 |
| 9 | Beit Jaan 1 | H | 93 183D | CRS | 153 195 263 | * |  |
| 10 | Beit Jaan 1 | H | CRS | 519 | 263 | R-P25 | 12 12 13 17 13 29 14 24 11 13 12 15 |
| 11 | Beit Jaan 1 | H | CRS | 519 | 263 | E-M35 | 11 12 11 16 13 31 13 23 10 13 10 14 |
| 12 | Beit Jaan 1 | H | 240 | 519 | 146 263 | E-M35 | 11 13 11 16 11 28 14 23 10 14 10 14 |
| 13 | Beq’ata 3 | H | 93 291 | 519 | 93 263 | E-M123 | 11 12 11 16 13 31 13 24 10 13 10 14 |
| 14 | Beq’ata 3 | H | CRS | 519 | 263 | * |  |
| 15 | Beq’ata 3 | H | 42 | 519 | 263 | E-M123 | 11 12 11 16 13 31 13 24 10 13 10 14 |
| 16 | Beq’ata 3 | H | 93 | 390 | 153 263 | J-M172 | 11 14 11 17 13 30 14 23 10 12 9 15 |
| 17 | Daliyat al-Karmel 2 | H | 42 | 519 | 263 | G-M201 | 11 12 11 15 12 29 15 22 11 13 10 16 |
| 18 | Daliyat al-Karmel 2 | H | 218 | 519 | 263 | R-P25 | 12 12 14 16 13 28 14 24 11 12 13 15 |
| 19 | Daliyat al-Karmel 2 | H | CRS | CRS | 263 | L-M20 | 11 12 14 16 13 30 15 22 11 11 10 15 |
| 20 | Daliyat al-Karmel 2 | H | 362 | 482 | 239 263 | L-M20 | 11 12 14 16 13 30 15 22 11 11 10 15 |
| 21 | Daliyat al-Karmel 2 | H | 187 218 | CRS | 263 | R-P25 | 12 12 13 16 13 29 14 24 10 14 12 15 |
| 22 | Daliyat al-Karmel 2 | H | 187 | CRS | 146 263 | E-M35 | 11 12 11 15 13 30 15 24 11 14 10 14 |
| 23 | Daliyat al-Karmel 2 | H | 93 265 | 519 | 263 | E-M35 | 11 12 11 17 13 30 13 24 10 13 10 14 |
| 24 | Daliyat al-Karmel 2 | H | 172 355 | 400 | 263 | E-M35 | 11 12 11 17 13 30 13 24 10 13 10 14 |
| 25 | Daliyat al-Karmel 2 | H | 114 356 | 519 | 263 | L-M20 | 11 12 14 16 13 30 15 22 11 11 10 15 |
| 26 | Hurfeish 1 | H | 265 | 519 | 263 | * |  |
| 27 | Hurfeish 1 | H | 93 | CRS | 73 153 195 263 | R-P25 | 12 12 13 17 13 29 14 24 11 13 12 15 |
| 28 | Jat 1 | H | 111 288 362 | CRS | 146 195 263 | J-M172 | 11 15 11 15 13 29 14 23 10 12 9 15 |
| 29 | Jat 1 | H | 111 288 362 | CRS | 146 195 263 | J-M172 | 11 15 11 14 13 29 14 23 10 12 9 15 |
| 30 | Jat 1 | H | 111 288 362 | CRS | 146 195 263 | J-M172 | 11 15 11 15 13 29 14 23 10 12 9 15 |
| 31 | Julis 1 | H | 93 | CRS | 153 195 263 | Q-P36 | 12 12 14 15 12 28 14 25 10 13 11 14 |
| 32 | Julis 1 | H | CRS | 519 | 93 263 | Q-P36 | 12 12 14 15 12 28 14 25 10 13 11 14 |
| 33 | Kisra 1 | H | 93 | 390 | 153 263 | G-M201 | 11 12 11 16 12 29 15 22 11 13 10 16 |
| 34 | Kisra 1 | H | 93 | 390 | 153 263 | G-M201 | 11 12 11 16 12 29 15 22 11 13 10 16 |
| 35 | Lebanon 4 | H | CRS | 519 | 263 | R-P25 | 12 12 13 17 13 29 14 24 11 13 12 15 |
| 36 | Lebanon 4 | H | 86 | 55.1 | 200 263 | R-P25 | 11 12 13 16 13 29 14 25 10 12 11 14 |
| 37 | Lebanon 4 | H | 93 | 390 | 153 263 | J-M267 | 11 17 11 15 13 29 15 23 11 12 10 14 |
| 38 | Lebanon 4 | H | CRS | CRS | 263 | J-M267 | 11 18 11 15 13 31 15 23 11 12 10 14 |
| 39 | Lebanon 4 | H | 93 265 | 519 | 263 | * |  |
| 40 | Lebanon 4 | H | 93 | 390 | 153 263 | J-M172 | 11 17 11 15 12 28 14 23 10 12 9 15 |
| 41 | Lebanon 4 | H | 187 | CRS | 146 263 | J-M172 | 11 16 11 15 14 29 14 22 9 12 9 15 |
| 42 | Lebanon 4-baysur | H | 93 | CRS | 153 195 263 | J-M267 | 11 17 11 15 13 30 15 23 11 12 10 14 |
| 43 | Lebanon 4-chatzbia | H | 93 | 519 | 263 | J-M172 | 11 15 11 15 14 29 14 22 9 12 9 16 |
| 44 | Lebanon 4-kfir | H | 93 | 390 | 153 263 | * |  |
| 45 | Lebanon 4-mari | H | 93 | CRS | 153 195 263 | J-M267 | 11 16 11 15 13 31 14 24 10 12 10 14 |
| 46 | Majdal Shams 3 | H | 188 | 519 | 263 | J-M267 | 11 17 11 15 13 30 14 23 10 12 10 14 |
| 47 | Majdal Shams 3 | H | CRS | 519 | 263 | * |  |
| 48 | Majdal Shams 3 | H | 362 | 482 | 239 263 | C-M216 | 11 13 11 15 14 30 16 23 10 14 10 14 |
| 49 | Majdal Shams 3 | H | 187 | CRS | 93 146 263 | J-M267 | 11 17 11 15 13 29 15 23 11 12 10 14 |
| 50 | Mughar 1 | H | 240 311 | 519 | 146 263 | G-M201 | 11 12 11 15 12 29 15 22 11 13 10 16 |
| 51 | Mughar 1 | H | CRS | 519 | 263 | E-M35 | 11 12 12 15 13 30 11 24 11 13 12 14 |
| 52 | Mughar 1 | H | CRS | 519 | 263 | E-M35 | 11 12 12 15 13 30 11 24 10 13 12 14 |
| 53 | Mughar 1 | H | 93 265 | 519 | 263 | K-M9 | 11 12 14 15 13 30 14 24 10 12 9 14 |
| 54 | Mughar 1 | H | 218 | 519 | 263 | R-M198 | 12 12 11 14 14 30 16 25 11 13 11 15 |
| 55 | Mughar 1 | H | CRS | 519 | 263 | J-M267 | 11 17 11 16 13 30 15 23 11 12 10 14 |
| 56 | Mughar 1 | H | 93 265 | 519 | 263 | J-M172 | 11 15 11 15 14 29 14 22 9 12 9 15 |
| 57 | Mughar 1 | H | CRS | 519 | 93 263 | J-M267 | 11 17 11 15 13 30 15 23 10 12 10 14 |
| 58 | Mughar 1 | H | 189 | 519 | 263 | J-M267 | 11 17 11 15 13 30 15 23 11 12 10 14 |
| 59 | Mughar 1 | H | CRS | 519 | 263 | E-M35 | 11 12 12 15 13 30 11 24 10 13 12 14 |
| 60 | Mughar 1 | H | 218 | 519 | 263 | J-M267 | 11 17 11 15 14 30 14 23 11 12 10 14 |
| 61 | Mughar 1 | H | 93 | CRS | 153 195 263 | * |  |
| 62 | Mughar 1 | H | 93 | CRS | 153 195 263 | * |  |
| 63 | Mughar 1 | H | 93 | CRS | 153 195 263 | * |  |
| 64 | Peq’in 1 | H | 93 291 | 519 | 93 263 | J-M172 | 11 15 11 15 14 29 14 22 9 12 9 15 |
| 65 | Peq’in 1 | H | 189 | 519 | 263 | J-M172 | 11 15 11 15 14 29 14 22 9 12 9 15 |
| 66 | Peq’in 1 | H | 218 | 519 | 263 | J-M267 | 11 17 11 15 13 30 15 23 12 12 10 14 |
| 67 | Rame 1 | H | CRS | 519 | 93 263 | J-M172 | 11 16 11 15 14 29 14 22 9 12 9 15 |
| 68 | Rame 1 | H | 93 | CRS | 153 195 263 | E-M123 | 11 12 11 17 13 31 13 24 10 13 10 14 |
| 69 | Rame 1 | H | 93 | CRS | 153 195 263 | G-M201 | 11 12 11 16 12 29 15 22 11 13 10 16 |
| 70 | Rame 1 | H | 218 | CRS | 263 | G-M201 | 11 12 11 15 12 29 15 22 11 13 10 16 |
| 71 | Rame 1 | H | 93 | CRS | 153 195 263 | R-P25 | 12 12 13 16 14 30 14 24 10 14 12 15 |
| 72 | Rame 1 | H | CRS | 519 | 263 | R-P25 | 12 12 13 17 13 29 14 24 10 13 12 15 |
| 73 | Sajur 1 | H | 42 257 | 519 | 263 | E-M35 | 11 12 11 17 13 30 13 24 10 13 10 14 |
| 74 | Sajur 1 | H | 293 304 | CRS | 153 263 | J-M172 | 11 17 11 15 12 28 14 23 10 12 9 15 |
| 75 | Shefar’am 1 | H | CRS | 519 | 146 195 263 | J-M267 | 11 17 11 16 14 30 14 23 11 12 10 14 |
| 76 | Shefar’am 1 | H | 93 291 | 519 | 93 263 | G-M201 | 11 12 11 16 14 32 15 22 10 14 10 16 |
| 77 | Shefar’am 1 | H | 187 | CRS | 146 263 | L-M20 | 11 12 13 17 14 30 14 23 10 12 10 16 |
| 78 | Shefar’am 1 | H | 320 | 519 | 263 | J-M267 | 11 17 11 16 14 31 14 23 11 12 10 14 |
| 79 | Sumei 1 | H | 189 | 519 | 263 | J-M267 | 11 17 11 15 13 29 15 23 11 12 10 14 |
| 80 | Sumei 1 | H | CRS | 519 | 263 | E-M35 | 11 13 11 16 11 28 14 23 10 14 10 14 |
| 81 | Syria 5 | H | 362 | 482 | 239 263 | E-M35 | 11 12 11 16 12 29 14 25 11 13 10 14 |
| 82 | Syria 5 | H | CRS | CRS | 153 195 263 | J-M267 | 11 16 11 15 14 31 14 23 10 12 10 14 |
| 83 | Syria 5 | H | CRS | CRS | 153 195 263 | J-M267 | 11 16 11 15 13 29 14 23 11 12 10 14 |
| 84 | Syria 5 | H | 189 | 519 | 263 | J-M267 | 11 18 11 15 13 30 15 23 10 12 10 14 |
| 85 | Syria 5 | H | 42 257 | 519 | 263 | J-M267 | 11 17 11 15 14 30 14 23 10 12 10 14 |
| 86 | Syria 5-kfir | H | 93 | 390 | 153 263 | R-M198 | 12 12 11 14 14 31 16 24 11 13 11 14 |
| 87 | Syria 5-Macheife | H | 42 | 519 | 263 | J-M172 | 11 15 11 15 14 29 14 22 9 12 9 15 |
| 88 | Syria 5-Salchat | H | CRS | 519 | 263 | R-P25 | 10 12 13 18 13 29 14 24 11 13 12 15 |
| 89 | Syria 5-urman | H | 93 | CRS | 153 195 263 | E-M123 | 11 12 11 16 14 32 14 24 10 14 10 14 |
| 90 | Isifya 2 | H | 42 | 519 | 263 | * |  |
| 91 | Isifya 2 | H | 42 | 519 | 263 | E-M123 | 11 12 11 16 13 32 13 24 10 13 10 14 |
| 92 | Isifya 2 | H | 42 | 519 | 263 | J-M172 | 11 15 10 14 13 30 14 24 9 12 7 14 |
| 93 | Isifya 2 | H | 42 | 519 | 263 | 0 | 11 12 11 16 13 29 15 22 11 13 10 16 |
| 94 | Yanuh 1 | H | 93 291 | 519 | 93 263 | R-P25 | 12 12 13 17 14 30 14 24 11 13 12 15 |
| 95 | Yanuh 1 | H | CRS | 519 | 93 263 | E-M35 | 11 12 11 14 14 30 13 24 9 13 10 14 |
| 96 | Yanuh 1 | H | 93 291 | 519 | 93 263 | E-M35 | 11 12 11 14 14 30 13 24 9 13 10 14 |
| 97 | Yirka 1 | H | 42 257 | 519 | 263 | L-M20 | 11 12 13 16 14 30 14 23 10 12 10 16 |
| 98 | Yirka 1 | H | 93 | CRS | 153 195 263 | J-M267 | 11 17 11 15 13 31 15 23 11 12 10 14 |
| 99 | Yirka 1 | H | 42 257 | 519 | 263 | J-M267 | 11 17 11 15 13 29 15 23 11 12 10 14 |
| 100 | Beq’ata 3 | HV | 67 234 | CRS | 152 263 | E-M123 | 11 12 11 16 13 31 13 24 10 13 10 14 |
| 101 | Beq’ata 3 | HV | 67 234 | CRS | 152 263 | * |  |
| 102 | Hurfeish 1 | HV | 67 131 292 354 | CRS | 183 263 | Q-P36 | 12 13 13 16 14 30 13 25 10 14 11 15 |
| 103 | Hurfeish 1 | HV | 67 131 292 354 | CRS | 183 263 | E-M123 | 11 12 11 15 13 30 15 23 10 14 11 14 |
| 104 | Hurfeish 1 | HV | 67 234 | CRS | 152 263 | E-M35 | 11 13 11 16 11 28 14 23 10 14 10 14 |
| 105 | Hurfeish 1 | HV | 67 | CRS | 150 263 | E-M35 | 11 13 11 16 11 28 14 23 10 14 10 14 |
| 106 | Julis 1 | HV | 67 | CRS | 150 263 | Q-P36 | 12 12 14 15 12 28 14 25 10 13 11 14 |
| 107 | Lebanon 4 | HV | 67 234 | CRS | 152 263 | K-M9 | 11 13 13 16 13 29 13 23 10 13 9 15 |
| 108 | Lebanon 4 | HV | 67 234 | CRS | 152 263 | * |  |
| 109 | Lebanon 4-nicha | HV | 67 234 | CRS | 152 263 | R-P25 | 12 12 13 15 14 30 14 24 10 14 12 15 |
| 110 | Majdal Shams 3 | HV | 67 | CRS | 150 263 | E-M35 | 11 12 11 16 13 31 14 23 10 13 10 14 |
| 111 | Yanuh 1 | HV | 67 | CRS | 150 263 | J-M267 | 11 17 11 15 13 31 15 23 12 12 10 14 |
| 112 | Yirka 1 | HV | 67 131 292 354 | CRS | 183 263 | K-M9 | 11 12 13 15 13 30 15 23 10 13 9 14 |
| 113 | Yirka 1 | HV | 67 131 292 354 | CRS | 183 263 | R-P25 | 12 12 13 16 13 29 14 24 11 12 12 15 |
| 114 | Yirka 1 | HV | 67 | CRS | 150 263 | E-M35 | 11 12 11 17 13 31 13 23 10 13 10 14 |
| 115 | Beit Jaan 1 | I | 129 223 320 | 391 519 | 73 199 204 250 263 291.1 | R-P25 | 12 12 13 17 13 30 14 24 11 13 12 15 |
| 116 | Beit Jaan 1 | I | 129 188 223 242 311 | 391 519 | 73 152 199 204 250 263 | E-M123 | 11 12 11 17 13 30 15 24 10 13 10 14 |
| 117 | Beq’ata 3 | I | 129 148 223 | 391 519 | 73 199 204 250 263 | E-M123 | 11 12 11 16 13 31 13 24 10 13 10 14 |
| 118 | Jat 1 | I | 129 223 320 | 391 519 | 73 199 204 250 263 291.1 | J-M172 | 11 15 10 14 13 30 14 23 9 12 7 14 |
| 119 | Jat 1 | I | 129 223 | 391 519 | 73 199 204 250 263 | J-M267 | 11 17 11 15 13 31 15 23 11 12 10 14 |
| 120 | Majdal Shams 3 | I | 129 223 | 391 519 | 73 199 204 250 263 | J-M172 | 11 16 11 15 14 29 14 22 9 12 9 15 |
| 121 | Rame 1 | I | 129 188 223 242 311 | 391 519 | 73 152 199 204 250 263 | G-M201 | 11 12 11 15 12 29 15 22 11 13 10 16 |
| 122 | Syria 5-schaita | I | 129 148 223 | 391 519 | 73 199 204 250 263 | * |  |
| 123 | Syria 5-schaita | I | 129 148 223 | 391 519 | 73 199 204 250 263 | J-M172 | 11 15 11 15 14 29 14 22 9 12 9 16 |
| 124 | Syria 5-schaita | I | 129 148 223 | 391 519 | 73 199 204 250 263 | J-M267 | 11 16 11 16 13 29 14 23 10 12 10 14 |
| 125 | Yirka 1 | I | 129 223 | 391 519 | 73 199 204 250 263 | E-M123 | 11 12 11 16 14 32 12 24 10 14 10 14 |
| 126 | Daliyat al-Karmel 2 | J | 69 126 145 261 290 | 519 | 73 195 263 271 295 | R-P25 | 12 12 13 16 13 29 14 24 10 14 12 15 |
| 127 | Ein Qiniyye 3 | J | 69 126 145 182C 183C 189 231 261 | CRS | 73 150 152 195 198 215 263 295 | R-P25 | 12 12 13 17 14 30 14 24 11 13 12 15 |
| 128 | Jordan-zarka 5 | J | 69 126 145 182C 183C 189 231 261 | CRS | 73 150 152 195 198 215 263 295 | G-M201 | 11 12 11 16 13 29 15 22 11 13 10 16 |
| 129 | Majdal Shams 3 | J | 69 126 145 182C 183C 189 231 261 | CRS | 73 150 152 195 198 215 263 295 | J-M267 | 11 17 11 15 13 30 14 23 10 12 10 14 |
| 130 | Majdal Shams 3 | J | 69 126 | 527 | 73 146 185 189 228 263 295 | E-M35 | 11 13 11 17 11 28 14 23 10 14 10 14 |
| 131 | Majdal Shams 3 | J | 69 126 145 182C 183C 189 231 261 | CRS | 73 150 152 195 198 215 263 295 | 0 | 11 13 12 15 13 29 16 23 10 14 10 14 |
| 132 | Majdal Shams 3 | J | 69 126 | 527 | 73 146 185 189 228 263 295 | J-M172 | 11 14 11 17 13 30 14 23 10 12 9 15 |
| 133 | Peq’in 1 | J | 69 126 145 261 290 | 519 | 73 195 263 271 295 | E-M2 | 11 12 11 16 13 30 16 21 10 14 11 14 |
| 134 | Sajur 1 | J | 69 126 | 527 | 73 146 185 189 228 263 295 | K-M9 | 11 12 14 15 13 29 14 24 10 12 9 14 |
| 135 | Syria 5 | J | 69 126 145 182C 183C 189 231 261 | CRS | 73 150 152 195 198 215 263 295 | * |  |
| 136 | Syria 5-Ira | J | 69 126 | CRS | 73 263 295 | J-M172 | 11 16 11 15 13 28 14 22 9 12 9 15 |
| 137 | Syria 5-kunetra | J | 69 126 214 | 527 | 73 146 185 189 228 263 295 | E-M35 | 11 12 11 16 12 29 13 24 10 13 10 14 |
| 138 | Isifya 2 | J | 69 126 145 261 290 | 519 | 73 195 263 271 295 | G-M201 | 11 12 11 16 12 29 15 22 11 13 10 16 |
| 139 | Isifya 2 | J | 69 126 145 261 290 | 519 | 73 195 263 271 295 | R-P25 | 12 12 13 16 13 29 14 24 11 12 12 15 |
| 140 | Yirka 1 | J | 69 126 | 527 | 73 146 185 189 228 263 295 | J-M172 | 11 15 10 14 13 30 14 23 9 12 7 14 |
| 141 | Beit Jaan 1 | K | 224 311 | 519 | 73 94 263 | E-M35 | 11 12 11 16 13 31 13 23 10 13 10 14 |
| 142 | Beit Jaan 1 | K | 167 216 224 311 368 | 519 | 73 146 195 263 | K-M9 | 11 12 13 15 14 31 14 23 10 13 9 14 |
| 143 | Beit Jaan 1 | K | 224 311 | 519 | 73 94 263 | E-M35 | 11 12 11 16 13 31 13 23 10 13 10 14 |
| 144 | Beq’ata 3 | K | 224 311 | 519 | 73 263 280G | J-M267 | 11 17 11 15 13 30 14 23 10 12 10 14 |
| 145 | Daliyat al-Karmel 2 | K | 93 224 311 362 | 519 | 73 263 | L-M20 | 11 14 14 16 13 30 15 23 11 11 10 15 |
| 146 | Daliyat al-Karmel 2 | K | 224 311 | 519 527 | 73 263 | E-M35 | 11 12 11 17 13 30 13 24 10 13 10 14 |
| 147 | Daliyat al-Karmel 2 | K | 224 311 | 519 527 | 73 263 | L-M20 | 11 12 14 16 13 30 15 22 11 11 10 15 |
| 148 | Daliyat al-Karmel 2 | K | 93 224 311 362 | 519 | 73 263 | L-M20 | 11 12 14 16 13 30 15 22 11 11 10 15 |
| 149 | Daliyat al-Karmel 2 | K | 224 311 | 519 | 73 263 280G | G-M201 | 11 12 12 15 12 29 14 23 11 13 10 15 |
| 150 | Daliyat al-Karmel 2 | K | 93 224 311 362 | 519 | 73 263 | R-M198 | 12 12 11 14 14 31 16 25 10 13 11 14 |
| 151 | Daliyat al-Karmel 2 | K | 224 311 | 519 527 | 73 263 | * |  |
| 152 | Daliyat al-Karmel 2 | K | 224 311 | 519 527 | 73 263 | * |  |
| 153 | Daliyat al-Karmel 2 | K | 93 224 311 362 | 519 | 73 263 | J-M267 | 11 13 11 14 15 31 14 23 10 12 10 14 |
| 154 | Hurfeish 1 | K | 93 111 224 311 | 519 | 73 263 | E-M35 | 11 13 11 17 11 28 14 23 10 14 10 14 |
| 155 | Hurfeish 1 | K | 224 239 260 311 | 519 k | 73 247 263 | R-P25 | 12 12 13 17 13 29 14 24 11 13 12 15 |
| 156 | Julis 1 | K | 167 216 224 311 368 | 519 | 73 146 195 263 | Q-P36 | 12 12 14 15 12 28 14 25 10 13 11 14 |
| 157 | Julis 1 | K | 167 216 224 311 368 | 519 | 73 146 195 263 | J-M267 | 11 17 11 15 13 30 14 23 10 12 10 14 |
| 158 | Julis 1 | K | 167 216 224 311 368 | 519 | 73 146 195 263 | K-M9 | 11 12 14 15 13 29 14 24 10 12 9 14 |
| 159 | Julis 1 | K | 167 216 224 311 368 | 519 | 73 146 195 263 | J-M172 | 11 14 11 15 12 28 14 23 10 12 9 15 |
| 160 | Kisra 1 | K | 167 216 224 311 368 | 519 | 73 146 195 263 | G-M201 | 11 12 11 16 12 29 15 22 11 13 10 16 |
| 161 | Lebanon 4 | K | 158 224 294 311 | 519 | 73 263 | E-M35 | 11 12 11 14 14 30 13 24 9 14 10 14 |
| 162 | Lebanon 4-ein jarfa | K | 224 311 | 519 | 73 263 280G | J-M267 | 11 17 11 15 13 30 14 23 10 12 10 14 |
| 163 | Majdal Shams 3 | K | 167 216 224 311 368 | 519 | 73 146 195 263 | J-M172 | 11 14 11 17 13 30 14 24 10 12 9 15 |
| 164 | Majdal Shams 3 | K | 224 311 | 519 | 73 263 280G | * |  |
| 165 | Mas’ade 3 | K | 93 224 311 | 519 | 73 263 | * |  |
| 166 | Peq’in 1 | K | 167 216 224 311 368 | 519 | 73 146 195 263 | E-M35 | 11 13 11 15 11 28 14 23 10 14 10 14 |
| 167 | Peq’in 1 | K | 167 216 224 311 368 | 519 | 73 146 195 263 | J-M172 | 11 15 10 14 13 30 14 23 9 12 7 14 |
| 168 | Rame 1 | K | 167 216 224 311 368 | 519 | 73 146 195 263 | G-M201 | 11 12 11 16 12 28 15 22 11 13 10 16 |
| 169 | Sajur 1 | K | 167 216 224 311 368 | 519 | 73 146 195 263 | J-M172 | 11 14 11 14 13 30 14 23 10 12 9 15 |
| 170 | Sajur 1 | K | 167 216 224 311 368 | 519 | 73 146 195 263 | K-M9 | 11 12 14 15 13 30 14 24 10 12 9 14 |
| 171 | Sajur 1 | K | 167 216 224 311 368 | 519 | 73 146 195 263 | K-M9 | 11 12 14 15 13 30 14 24 10 12 9 14 |
| 172 | Sajur 1 | K | 167 216 224 311 368 | 519 | 73 146 195 263 | K-M9 | 11 12 14 15 13 30 14 24 10 12 9 14 |
| 173 | Shefar’am 1 | K | 167 216 224 311 368 | 519 | 73 146 195 263 | L-M20 | 11 12 13 16 13 29 14 23 10 12 10 16 |
| 174 | Syria 5 | K | 158 224 294 311 | 519 | 73 263 | J-M267 | 11 17 11 15 13 30 15 23 10 12 10 14 |
| 175 | Syria 5-schaita | K | 93 111 224 311 | 519 | 73 263 | J-M267 | 11 17 11 15 13 29 15 23 11 12 10 14 |
| 176 | Isifya 2 | K | 224 311 362 | 519 | 73 263 | 0 | 11 17 11 15 14 30 14 23 11 12 10 14 |
| 177 | Isifya 2 | K | 158 224 294 311 | 519 | 73 263 | E-M35 | 11 13 11 16 11 28 14 23 10 14 10 14 |
| 178 | Yirka 1 | K | 167 216 224 311 368 | 519 | 73 146 195 263 | E-M123 | 11 12 11 16 14 32 12 24 10 14 10 14 |
| 179 | Yirka 1 | K | 167 216 224 311 368 | 519 | 73 146 195 263 | J-M172 | 11 17 11 15 12 28 14 23 10 12 9 15 |
| 180 | Beit Jaan 1 | L2a3 | 189 192 223 278 292 294 309 | 390 519 | 73 143 146 151 152 195 263 | R-P25 | 12 12 13 17 13 29 14 24 11 13 12 15 |
| 181 | Daliyat al-Karmel 2 | L2a3 | 189 192 223 278 292 294 309 | 390 519 | 73 143 146 152 195 263 | G-M201 | 11 12 11 15 12 29 15 22 11 13 10 16 |
| 182 | Daliyat al-Karmel 2 | L2a3 | 189 192 223 278 292 294 309 | 390 519 | 73 143 146 152 195 263 | L-M20 | 11 12 14 16 13 29 14 22 10 11 10 14 |
| 183 | Lebanon 4 | L2a3 | 189 192 223 278 292 294 309 | 390 519 | 73 143 146 152 195 263 | J-M267 | 11 17 11 15 14 30 14 23 11 12 10 14 |
| 184 | Lebanon 4-shuafat | L2a3 | 189 192 223 278 292 294 309 | 390 519 | 73 143 146 152 195 263 | G-M201 | 11 12 11 16 13 29 15 22 11 13 10 16 |
| 185 | Mughar 1 | L2a3 | 189 192 223 278 292 294 | 390 519 | 73 143 146 152 195 263 | E-M35 | 11 12 11 16 12 30 14 25 11 13 10 14 |
| 186 | Isifya 2 | L2a3 | 189 192 223 278 292 294 309 | 390 519 | 73 143 146 152 195 263 | R-P25 | 12 12 13 16 13 29 14 24 11 12 12 15 |
| 187 | Beit Jaan 1 | M1 | 129 189 223 249 311 359 | 519 | 73 195 263 | J-M172 | 11 15 11 14 13 30 14 23 9 12 7 14 |
| 188 | Jat 1 | M1 | 129 183C 189 223 249 311 359 | 519 | 73 195 263 | J-M172 | 11 15 11 15 13 29 14 23 10 12 9 15 |
| 189 | Jat 1 | M1 | 129 183C 189 223 249 311 359 | 519 | 73 195 263 | R-P25 | 12 12 13 16 13 29 14 24 11 12 12 15 |
| 190 | Kisra 1 | M1 | 129 189 223 249 311 359 | 519 | 73 195 263 | G-M201 | 11 12 11 16 12 29 15 22 11 13 10 16 |
| 191 | Lebanon 4 | M1 | 129 189 223 249 311 359 | 519 | 73 195 263 | J-M172 | 11 13 11 14 13 30 14 23 9 12 7 14 |
| 192 | Kisra 1 | N1 | 93 223 265 | 519 | 73 189 195 204 210 263 | K-M9 | 11 12 11 17 13 29 15 22 9 13 9 14 |
| 193 | Beit Jaan 1 | N1B | 145 176G 223 | 390 519 | 73 152 263 | J-M172 | 11 15 11 14 13 30 14 23 9 12 7 14 |
| 194 | Beq’ata 3 | N1B | 145 176G 223 362 | 390 519 | 73 152 263 | J-M267 | 11 17 11 15 13 29 15 24 11 12 10 14 |
| 195 | Hurfeish 1 | N1B | 145 176G 223 | 390 519 | 73 152 263 | R-P25 | 12 12 13 17 13 29 14 24 11 13 12 15 |
| 196 | Mughar 1 | N1B | 51 145 176G 223 | 390 519 | 73 152 263 | E-M35 | 11 12 11 16 13 30 14 24 11 13 10 14 |
| 197 | Peq’in 1 | N1B | 93 129 145 176G 223 | 390 519 | 73 152 263 | J-M172 | 11 15 11 15 14 29 14 22 9 12 9 15 |
| 198 | Peq’in 1 | N1B | 145 176G 223 | 390 519 | 73 152 263 | J-M172 | 11 15 11 15 14 29 14 22 9 12 9 15 |
| 199 | Peq’in 1 | N1B | 93 129 145 176G 223 | 390 519 | 73 152 263 | J-M267 | 11 17 11 15 13 31 15 23 12 12 10 14 |
| 200 | Beit Jaan 1 | preHV | 126 362 | CRS | 58.1 64 263 | J-M267 | 11 12 11 15 13 29 14 23 11 12 10 14 |
| 201 | Daliyat al-Karmel 2 | preHV | 217 | 519 | 73 152 263 | K-M9 | 11 12 13 15 14 31 14 22 10 13 9 14 |
| 202 | Hurfeish 1 | preHV | 114 126 274 362 | 519 | 57.1 64 143 263 | E-M123 | 11 12 11 17 13 31 13 24 10 13 10 14 |
| 203 | Julis 1 | preHV | 126 255 355 362 | CRS | 58 64 146 152 195 263 279 | G-M201 | 11 12 11 15 12 29 15 22 11 13 10 16 |
| 204 | Lebanon 4 | preHV | 126 255 355 362 | CRS | 58 64 146 152 195 263 279 | G-M201 | 11 12 11 15 12 29 15 22 11 13 10 16 |
| 205 | Mughar 1 | preHV | 93 126 362 | 482 | 58.1 64 263 279 | R-P25 | 10 12 13 17 13 29 14 24 10 13 12 15 |
| 206 | Mughar 1 | preHV | 126 189 362 | CRS | 58.1 64 263 | E-M35 | 11 12 11 16 13 31 13 23 10 13 10 14 |
| 207 | Syria 5 | preHV | 126 183C 189 293 362 | CRS | 58.1 64 263 | R-P25 | 12 12 13 15 14 30 14 24 10 14 12 15 |
| 208 | Yanuh 1 | preHV | 114 126 362 | 519 | 57.1 64 143 263 | E-M35 | 11 12 11 14 14 30 13 24 9 13 10 14 |
| 209 | Syria 5 | preHV | 311 | CRS | 152 185 263 | J-M267 | 11 17 11 15 13 30 14 23 10 12 10 14 |
| 210 | Beq’ata 3 | preV | 298 | CRS | 72 152 195 263 | * |  |
| 211 | Beq’ata 3 | preV | 298 | CRS | 72 152 195 263 | * |  |
| 212 | Abu Sinan 1 | T | 126 163 186 189 294 | 519 | 73 150 263 | G-M201 | 11 12 11 16 13 29 15 22 11 13 10 16 |
| 213 | Beit Jaan 1 | T | 126 294 | CRS | 73 263 | E-M35 | 11 12 11 16 13 31 13 23 10 13 10 14 |
| 214 | Beit Jaan 1 | T | 126 294 | CRS | 73 263 | J-M267 | 11 17 11 15 13 31 14 23 10 12 10 14 |
| 215 | Beit Jaan 1 | T | 126 294 | CRS | 73 263 | R-P25 | 12 12 13 17 13 29 14 24 11 13 12 15 |
| 216 | Beit Jaan 1 | T | 126 294 | CRS | 73 263 | R-P25 | 12 12 13 17 13 29 14 24 11 13 12 15 |
| 217 | Beit Jaan 1 | T | 126 294 | CRS | 73 263 | R-P25 | 12 12 13 17 13 29 14 24 11 13 12 13 |
| 218 | Daliyat al-Karmel 2 | T | 126 163 186 189 291 294 | CRS | 73 152 263 | R-P25 | 12 12 13 16 13 29 14 24 11 12 12 15 |
| 219 | Kisra 1 | T | 126 140 189 294 296 311 | 519 | 73 263 | G-M201 | 11 12 11 16 12 29 15 22 11 13 10 16 |
| 220 | Lebanon 4 | T | 126 163 186 189 291 294 | CRS | 73 152 263 | J-M267 | 11 16 12 15 13 30 14 23 11 12 10 14 |
| 221 | Lebanon 4 | T | 126 163 186 189 294 | 519 | 73 150 263 | L-M20 | 11 12 14 16 13 30 15 22 11 11 10 15 |
| 222 | Lebanon 4-baysur | T | 126 163 172 186 189 294 298 | 399 519 | 73 151 263 | J-M172 | 11 14 11 16 14 31 14 24 10 12 9 15 |
| 223 | Majdal Shams 3 | T | 126 163 186 189 294 | 519 | 73 150 263 | E-M123 | 11 12 11 16 14 32 12 24 10 14 10 14 |
| 224 | Majdal Shams 3 | T | 126 163 186 189 294 | 519 | 73 150 263 | * |  |
| 225 | Majdal Shams 3 | T | 126 163 186 189 294 | 519 | 73 150 263 | G-M201 | 11 12 11 15 12 29 15 22 11 13 10 16 |
| 226 | Majdal Shams 3 | T | 126 163 186 189 294 | 519 | 73 150 263 | G-M201 | 11 12 11 16 13 30 15 22 10 13 10 16 |
| 227 | Majdal Shams 3 | T | 126 163 186 189 294 | 519 | 73 150 263 | J-M267 | 11 17 11 15 13 29 15 23 11 12 10 14 |
| 228 | Mas’ade 3 | T | 126 163 186 189 294 | 519 | 73 150 263 | 0 | 11 12 11 16 14 30 13 22 10 13 10 14 |
| 229 | Mughar 1 | T | 126 163 172 186 189 294 298 | 399 519 | 73 151 263 | K-M9 | 11 12 14 15 13 30 14 24 10 12 9 14 |
| 230 | Sumei 1 | T | 126 163 186 189 294 | 519 | 73 150 263 | J-M172 | 11 14 11 14 14 30 14 23 10 12 9 15 |
| 231 | Syria 5 | T | 126 294 304 311 | 519 | 73 146 263 | Q-P36 | 12 12 14 16 14 30 13 22 10 13 11 14 |
| 232 | Syria 5 | T | 126 163 186 189 294 | 519 | 73 150 214 263 | J-M267 | 11 17 11 15 13 30 15 23 11 12 10 14 |
| 233 | Syria 5 | T | 126 163 186 189 294 | 519 | 73 150 263 | 0 | 11 12 11 16 13 31 13 24 10 13 10 14 |
| 234 | Isifya 2 | T | 126 294 304 311 | 519 | 73 146 263 | E-M123 | 11 12 11 16 13 32 13 24 10 13 10 14 |
| 235 | Abu Sinan 1 | U | 192 256 270 311 | 526 | 73 263 | K-M9 | 11 12 13 16 14 31 14 23 10 13 9 14 |
| 236 | Abu Sinan 1 | U | 192 256 270 311 | 526 | 73 263 | G-M201 | 11 12 11 16 13 29 15 22 11 13 10 16 |
| 237 | Beit Jaan 1 | U | 183C 189 249 261 | CRS | 73 263 285 | R-P25 | 12 12 13 16 13 29 14 24 11 13 12 15 |
| 238 | Beit Jaan 1 | U | 173 260 343 | 390 | 73 150 199 263 | R-P25 | 12 12 13 17 13 29 14 24 11 13 12 15 |
| 239 | Beit Jaan 1 | U | 249 311 327 | CRS | 73 146 152 195 263 285 | R-P25 | 12 12 13 17 13 29 14 24 11 13 12 15 |
| 240 | Beit Jaan 1 | U | 183C 189 249 261 | CRS | 73 263 285 | R-P25 | 12 12 13 16 14 30 14 24 10 14 12 15 |
| 241 | Beq’ata 3 | U | 249 311 327 | CRS | 73 146 152 195 263 285 | E-M123 | 11 12 11 17 13 31 13 24 10 13 10 14 |
| 242 | Beq’ata 3 | U | 249 311 327 | CRS | 73 146 152 195 263 285 | J-M267 | 11 17 11 15 13 30 15 24 11 12 10 14 |
| 243 | Beq’ata 3 | U | 249 311 327 | CRS | 73 146 152 195 263 285 | J-M267 | 11 17 11 15 13 30 14 23 10 12 10 14 |
| 244 | Lebanon 4 | U | 249 311 327 | CRS | 73 146 152 195 263 285 | G-M201 | 11 12 11 15 12 29 15 22 11 13 10 16 |
| 245 | Lebanon 4 | U | 51 129T 183C 189 243 362 | 519 | 73 152 217 263 | * |  |
| 246 | Lebanon 4-baysu | U | 51 129T 183C 189 243 362 | 519 | 73 152 217 263 | R-P25 | 11 12 13 15 13 29 14 25 10 12 11 15 |
| 247 | Lebanon 4-mimas | U | 249 311 327 | CRS | 73 146 152 195 263 285 | E-M123 | 11 12 11 17 13 31 13 24 10 13 10 14 |
| 248 | Majdal Shams 3 | U | 168 192 223 343 | CRS | 73 150 263 | G-M201 | 11 12 11 17 13 31 15 22 10 14 10 16 |
| 249 | Majdal Shams 3 | U | 111 172 189 234 311 | 519 | 73 195 263 | I-M223 | 11 13 12 15 13 29 16 23 10 14 10 14 |
| 250 | Majdal Shams 3 | U | 111 172 189 234 311 | 519 | 73 195 263 | J-M172 | 11 14 11 17 13 29 14 23 10 12 9 15 |
| 251 | Mughar 1 | U | 249 311 327 | CRS | 73 146 152 195 263 285 | J-M267 | 11 16 11 15 13 30 14 23 10 12 10 14 |
| 252 | Mughar 1 | U | 183C 189 249 261 | CRS | 73 263 285 | K-M9 | 11 12 13 15 14 31 14 23 10 13 9 14 |
| 253 | Peq’in 1 | U | 183C 189 249 261 | CRS | 73 263 285 | R-P25 | 12 12 13 16 14 32 14 24 10 14 12 15 |
| 254 | Peq’in 1 | U | 183C 189 249 261 | CRS | 73 263 285 | L-M20 | 11 12 14 17 12 28 14 23 10 11 10 15 |
| 255 | Rame 1 | U | 93 145 309 318T | 519 | 73 152 153 263 | G-M201 | 11 12 11 15 12 29 15 22 11 13 10 16 |
| 256 | Rame 1 | U | 93 145 309 318T | 519 51 | 73 152 153 263 | G-M201 | 11 12 11 15 12 29 15 22 12 13 10 16 |
| 257 | Rame 1 | U | 227 309 318T | 519 | 73 152 263 | G-M201 | 11 12 11 15 12 29 15 22 11 13 10 16 |
| 258 | Shefar’am 1 | U | 249 311 327 | CRS | 73 146 152 195 263 285 | R-M124 | 12 12 10 15 14 30 14 23 11 13 11 16 |
| 259 | Sumei 1 | U | 249 311 327 | CRS | 73 146 152 195 263 285 | J-M172 | 11 16 11 15 14 29 14 22 9 12 9 15 |
| 260 | Sumei 1 | U | 249 311 327 | CRS | 73 146 152 195 263 285 | E-M35 | 11 12 11 14 14 30 13 23 9 14 10 14 |
| 261 | Syria 5-swieda | U | 249 311 327 | CRS | 73 146 152 195 263 285 | K-M9 | 11 12 14 15 13 30 14 24 10 12 9 14 |
| 262 | Syria 5-swieda | U | 192 256 270 311 | 526 | 73 263 | E-M35 | 11 12 11 16 12 29 14 25 11 13 10 14 |
| 263 | Isifya 2 | U | 37 309 318T | 519 | 73 151 152 263 | R-P25 | 12 12 13 16 13 29 14 24 11 12 12 15 |
| 264 | Yanuh 1 | U | 249 311 327 | CRS | 73 146 152 195 263 285 | R-P25 | 12 12 13 17 13 28 14 24 10 13 12 15 |
| 265 | Yanuh 1 | U | 249 311 327 | CRS | 73 146 152 195 263 285 | J-M172 | 11 15 11 15 14 29 14 22 9 12 9 15 |
| 266 | Yirka 1 | U | 111 172 189 234 311 | 519 | 73 195 263 | J-M267 | 11 15 11 15 13 30 15 25 11 13 10 14 |
| 267 | Mughar 1 | W | 223 292 | 519 | 73 189 194 195 199 204 207 263 | J-M172 | 11 15 11 15 14 29 15 22 9 12 9 15 |
| 268 | Mughar 1 | W | 223 292 | 519 | 73 183 189 195 204 207 263 | J-M267 | 11 17 11 15 13 30 15 23 11 12 10 14 |
| 269 | Syria 5 | W | 223 292 295 | 519 | 73 143 189 194 195 199 204 207 | G-M201 | 11 12 11 16 12 29 15 22 11 13 10 16 |
| 270 | Isifya 2 | W | 223 292 | 519 | 73 189 194 195 199 204 207 263 | J-M172 | 11 15 10 14 13 30 14 23 9 12 7 14 |
| 271 | Beit Jaan 1 | X | 104 145 182C 183C 189 223 | 519 | 73 146 153 263 | K-M9 | 11 12 13 15 14 31 14 23 10 13 9 14 |
| 272 | Beit Jaan 1 | X | 104 145 182C 183C 189 223 | 519 | 73 146 153 263 | J-M267 | 11 17 11 15 13 30 14 23 10 12 10 14 |
| 273 | Beit Jaan 1 | X | 104 145 182C 183C 189 223 | 519 | 73 146 153 263 | K-M9 | 11 12 13 15 14 31 14 23 10 13 9 14 |
| 274 | Beit Jaan 1 | X | 104 145 182C 183C 189 223 | 519 | 73 146 153 263 | R-P25 | 12 12 13 16 14 30 14 25 11 13 12 15 |
| 275 | Beit Jaan 1 | X | 104 145 182C 183C 189 223 | 519 | 73 146 153 263 | R-P25 | 12 12 13 16 13 29 14 24 11 13 12 15 |
| 276 | Beit Jaan 1 | X | 104 145 182C 183C 189 223 | 519 | 73 146 153 263 | J-M267 | 11 17 11 15 13 30 14 23 10 12 10 14 |
| 277 | Beit Jaan 1 | X | 104 145 182C 183C 189 223 | 519 | 73 146 153 263 | K-M9 | 11 12 13 15 14 31 14 23 10 13 9 14 |
| 278 | Beit Jaan 1 | X | 189 278 311 | 519 | 73 153 195 225 263 | J-M267 | 11 17 11 15 13 29 15 23 11 12 10 14 |
| 279 | Beit Jaan 1 | X | 104 145 182C 183C 189 223 | 519 | 73 146 153 263 | R-P25 | 12 12 13 17 13 30 14 24 11 13 12 15 |
| 280 | Beit Jaan 1 | X | 104 145 182C 183C 189 223 | 519 | 73 146 153 263 | G-M201 | 11 12 11 15 12 29 15 22 11 13 10 16 |
| 281 | Beit Jaan 1 | X | 189 278 | 519 | 73 153 195 225 263 | 0 | 11 12 11 16 14 30 13 22 10 13 10 14 |
| 282 | Hurfeish 1 | X | 104 145 182C 183C 189 223 | 519 | 73 146 263 | E-M35 | 11 13 11 16 11 28 14 23 10 14 10 14 |
| 283 | Jat 1 | X | 189 278 | 519 | 73 153 195 225 263 | G-M201 | 11 12 11 16 13 30 15 22 10 13 10 16 |
| 284 | Jat 1 | X | 189 278 | 519 | 73 153 195 225 263 | J-M172 | 11 15 11 15 13 29 14 23 10 12 9 15 |
| 285 | Julis 1 | X | 126 183C 189 223 278 | 519 | 73 146 153 195 256 263 | K-M9 | 11 12 13 15 14 31 14 23 10 13 9 14 |
| 286 | Julis 1 | X | 189 278 | 519 | 73 153 195 225 227 263 | Q-P36 | 12 12 14 15 12 28 14 25 10 13 11 14 |
| 287 | Julis 1 | X | 126 183C 189 223 278 | 519 | 73 146 153 195 256 263 | K-M9 | 11 12 13 15 14 31 14 23 10 13 9 14 |
| 288 | Julis 1 | X | 126 183C 189 223 278 | 519 | 73 146 153 256 263 | J-M267 | 11 17 11 15 13 31 14 23 11 12 10 14 |
| 289 | Kisra 1 | X | 189 278 | 519 | 73 153 195 225 263 | G-M201 | 11 12 11 16 12 28 15 22 11 13 10 16 |
| 290 | Kisra 1 | X | 189 278 | 519 | 73 153 195 225 263 | G-M201 | 11 12 11 16 12 29 15 22 11 13 10 16 |
| 291 | Lebanon 4 | X | 189 278 | 519 | 73 153 195 225 263 | K-M9 | 11 12 13 16 13 30 14 23 10 13 9 14 |
| 292 | Lebanon 4-hatzbia | X | 189 223 274 278 344 | 519 | 73 153 195 225 263 | J-M172 | 11 15 11 15 13 29 14 23 10 12 9 15 |
| 293 | Mughar 1 | X | 189 278 | 519 | 73 153 195 225 263 | R-M198 | 12 12 11 15 13 29 16 25 11 13 11 15 |
| 294 | Mughar 1 | X | 126 183C 189 223 278 | 519 | 73 153 195 225 226 263 | * |  |
| 295 | Mughar 1 | X | 126 183C 189 223 278 | 519 | 73 153 195 225 226 263 | * |  |
| 296 | Mughar 1 | X | 126 183C 189 223 278 | 519 | 73 153 195 225 226 263 | * |  |
| 297 | Mughar 1 | X | 126 183C 189 223 278 | 519 | 73 153 195 225 226 263 | * |  |
| 298 | Mughar 1 | X | 126 183C 189 223 278 | 519 | 73 153 195 225 226 263 | E-M35 | 11 12 12 15 13 30 11 24 10 13 12 14 |
| 299 | Peq’in 1 | X | 126 183C 189 223 278 | 519 | 73 146 153 256 263 | J-M172 | 11 13 11 15 14 29 14 22 9 12 9 15 |
| 300 | Peq’in 1 | X | 189 278 | 519 | 73 153 195 225 263 | J-M172 | 11 15 11 15 14 29 14 22 9 12 9 15 |
| 301 | Peq’in 1 | X | 183C 189 223 278 311 | 519 10 | 73 153 195 263 | J-M172 | 11 15 11 15 14 29 14 22 9 12 9 15 |
| 302 | Peq’in 1 | X | 104 145 182C 183C 189 223 | 519 | 73 146 153 263 | G-M201 | 11 12 11 15 12 29 15 22 10 13 10 16 |
| 303 | Peq’in 1 | X | 104 145 182C 183C 189 223 | 519 | 73 146 153 263 | * |  |
| 304 | Peq’in 1 | X | 189 278 | 519 | 73 153 195 225 263 | E-M35 | 11 13 11 15 11 28 14 23 11 14 10 14 |
| 305 | Sajur 1 | X | 126 189 223 278 | 519 | 73 153 195 257 263 | K-M9 | 11 12 14 15 13 30 14 24 10 12 9 14 |
| 306 | Shefar’am 1 | X | 126 183C 189 223 278 | 519 | 73 146 153 256 263 | L-M20 | 11 12 14 16 12 28 14 22 10 11 10 14 |
| 307 | Sumei 1 | X | 126 183C 189 223 278 | 519 | 73 153 195 225 226 263 | * |  |
| 308 | Sumei 1 | X | 189 278 311 | 519 | 73 153 195 225 263 | R-P25 | 12 12 13 16 14 30 14 24 13 14 12 15 |
| 309 | Yirka 1 | X | 126 183C 189 223 278 | 519 | 73 153 195 225 226 263 | R-P25 | 12 12 13 16 13 29 14 24 11 12 12 15 |
| 310 | Yirka 1 | X | 126 183C 189 223 278 | 519 | 73 153 195 225 226 263 | K-M9 | 11 12 13 16 13 31 15 23 10 13 9 14 |
| 311 | Yirka 1 | X | 126 183C 189 223 278 | 519 | 73 153 195 225 226 263 | Q-P36 | 12 12 14 15 12 27 14 24 10 13 11 14 |

Maternal Origin: 1=Galilee; 2=Carmel; 3=Golan; 4=Lebanon ; 5=Syria .

Haplogroup Y: *= Females 0=have not been analyzed.

Summary Y STR order: *DYS426*, *DYS388, DYS392, DYS439 DYS389I, DYS389II, DYS19, DYS390, DYS391, DYS393, DYS438 and DYS457*.

Heteroplasmic mtDNA mutations have been ignored.
