# Supplementary material for: The Druze: A Population Genetic Refugium of the Near East
Source: PLoS One. 2008 May 7;3(5):e2105. doi: 10.1371/journal.pone.0002105 (PMC2324201; doi:10.1371/journal.pone.0002105)
Supplement: Table S3 — (0.04 MB DOC) [file pone.0002105.s003.doc]

**Table S3**: Druze Mitochondrial Haplogroup and Haplotype Frequencies.

| Haplogroup | # Haplotypes* | # Samples | Haplogroup Frequency % |
| --- | --- | --- | --- |
| H | 32 | 99 | 31.83 |
| HV | 3 | 15 | 4.82 |
| I | 4 | 11 | 3.54 |
| J | 5 | 15 | 4.82 |
| K | 10 | 39 | 12.54 |
| L2a3 | 3 | 7 | 2.25 |
| M1 | 2 | 5 | 1.61 |
| N1 | 1 | 1 | 0.32 |
| N1b | 4 | 7 | 2.25 |
| preHV | 9 | 10 | 3.22 |
| preV | 1 | 2 | 0.64 |
| T | 7 | 23 | 7.40 |
| U | 11 | 32 | 10.29 |
| W | 3 | 4 | 1.29 |
| X | 11 | 41 | 13.18 |
| Total | 106 | 311 | 100 |

*Haplotypes have been designated according to mitochondrial D-loop nucleotides 16024-00300.
