# Supplementary material for: The Druze: A Population Genetic Refugium of the Near East
Source: PLoS One. 2008 May 7;3(5):e2105. doi: 10.1371/journal.pone.0002105 (PMC2324201; doi:10.1371/journal.pone.0002105)
Supplement: Table S4 — (0.05 MB DOC) [file pone.0002105.s004.doc]

**Table S4: The probability (p-value) of obtaining the Druze X haplogroup frequency as a result of correlated sampling from Turks with the parameter *ρ*.**

| **Rho** | **N=20,000, n=500** | **N=50,000, n=500** | **N=20,000, n=1,000** | **N=50,000, n=1,000** |
| --- | --- | --- | --- | --- |
| **0** | < 0.001 | < 0.001 | < 0.001 | < 0.001 |
| **0.1** | < 0.001 | < 0.001 | < 0.001 | < 0.001 |
| **0.2** | < 0.001 | < 0.001 | < 0.001 | < 0.001 |
| **0.3** | < 0.001 | < 0.001 | < 0.001 | < 0.001 |
| **0.4** | 0.001 | 0.001 | < 0.001 | < 0.001 |
| **0.5** | 0.004 | 0.003 | 0.002 | < 0.001 |
| **0.6** | 0.013 | 0.011 | 0.011 | 0.012 |
| **0.7** | 0.029 | 0.023 | 0.031 | 0.029 |
| **0.8** | 0.04 | 0.045 | 0.037 | 0.044 |
| **0.9** | 0.032 | 0.031 | 0.043 | 0.046 |
| **1** | 0.031 | 0.025 | 0.033 | 0.034 |

In table S4 we have tested the hypothesis that the fraction of X haplogroup within the Druze population may have been a result of a non-random sampling due to correlation between immigrants. In our calculations we follow the method presented in Rothman et. al[1]. As possible reference source populations, we used nearby populations with the smallest sum square distance of haplogroup frequencies. The X haplogroup fraction in the Turkish population, the population with the smallest haplogroup frequency distance, is 6/218[2]. We then used numerical calculations to assess the probability of obtaining the high fraction of Druze X haplogroup individuals (41/311) as a result of non-random sampling of the population.

*N* denotes the size of the source population, *n* – the size of the derived new population, *f* – the frequency of the X haplogroup in the source population, and *ρ* – the correlation between sampled individuals.

Description of the simulation: For 1,000 iterations, we initiate a vector *V* of zeros of length *n*. *Vi*=1 if individual *i* is an X haplogroup, and 0 otherwise. We have used the arcsine transform method to estimate the X haplogroup fraction in the source population as$f=\sin(p^{2})$, where *p* is a random number drawn from a normal distribution with mean sqrt*(6/218)* and variance *1/(4*218)*. This accounts for the sampling uncertainty in the exact X halogroup fraction in the source population. We then proceed to assign the state of each new sampled individual. The first draw is an X haplogroup with probability *f*. There after, in the *i*th draw we flip a coin and with probability (1- *ρ*) set *Vi=1* with probability *f.* With probability *ρ* we set *Vi=1* with probability *f'* where *f'*=$(\sum_{j=1}^{i-1} V_{j})/(j-1)$. Therefore with probability *ρ* we set the haplogroup of the new *i*th individual as 1 according to the proportion of already existent X haplogroups in the sample so far. With probability 1- *ρ*, the new sampled individual is not correlated to the sample so far, and we set its state according to the probability of the X haplogroup in the source population, *f*.

When the vector *V* has been filled (after *n* steps), we count the number of X haplogroup individuals (number of ones in the vector *V*). Finally we compute the distribution of this value over the 1,000 iterations.

We have used the same simulations to calculate the probability of obtaining the Druze X haplogroup frequency as a result of correlated sampling from Egyptians, Armenians and other combinations of the three populations (Data not shown), and found p values<0.01 for the reported parameters of the simulation in table S4 (*N,n,rho*).

1. Rothman, E.D., C.F. Sing, and A.R. Templeton, A model for analysis of population structure. Genetics, 1974. 78(3): p. 943-60.

2. Macaulay, V., et al., The emerging tree of West Eurasian mtDNAs: a synthesis of control-region sequences and RFLPs. Am J Hum Genet, 1999. 64(1): p. 232-49.
