# Supplementary material for: The Druze: A Population Genetic Refugium of the Near East
Source: PLoS One. 2008 May 7;3(5):e2105. doi: 10.1371/journal.pone.0002105 (PMC2324201; doi:10.1371/journal.pone.0002105)
Supplement: Table S5 — (0.04 MB DOC) [file pone.0002105.s005.doc]

**Table S5: Druze Nei's Genetic Diversity in mtDNA and NRY-STR haplotypes**

|  | Carmel | Galilee | Golan | Lebanon | Syria |
| --- | --- | --- | --- | --- | --- |
| mtDNA # Haplotypes | 21 | 73 | 22 | 21 | 22 |
| # Samples | 35 | 184 | 37 | 29 | 26 |
| H^* (mtDNA) | 0.953 | 0.976 | 0.917 | 0.961 | 0.995 |
| STR # Haplotypes | 22 | 64 | 17 | 12 | 23 |
| # Samples | 33 | 183 | 24 | 12 | 28 |
| H^* (STR) | 0.955 | 0.968 | 0.924 | 1.000 | 0.987 |

*****H= Nei's Genetic diversity[1].

1. Nei, M.K., S., Molecular Evolution and and Phylogenetics P. 245. 2000: Oxford University Press
