# Supplementary material for: The Druze: A Population Genetic Refugium of the Near East
Source: PLoS One. 2008 May 7;3(5):e2105. doi: 10.1371/journal.pone.0002105 (PMC2324201; doi:10.1371/journal.pone.0002105)
Supplement: Table S6 — (0.04 MB DOC) [file pone.0002105.s006.doc]

**Table S6: Druze NRY Haplogroup Frequencies.**

| Region\  Haplogroup | Carmel | Galilee | Golan | Lebanon | Syria |
| --- | --- | --- | --- | --- | --- |
| C | 0.00 | 0.00 | 0.04 | 0.00 | 0.00 |
| E | 0.15 | 0.18 | 0.29 | 0.00 | 0.29 |
| G | 0.12 | 0.14 | 0.04 | 0.00 | 0.14 |
| I | 0.00 | 0.00 | 0.08 | 0.00 | 0.00 |
| J | 0.18 | 0.31 | 0.54 | 0.58 | 0.39 |
| K | 0.00 | 0.11 | 0.00 | 0.17 | 0.04 |
| L | 0.27 | 0.02 | 0.00 | 0.08 | 0.00 |
| Q | 0.00 | 0.04 | 0.00 | 0.08 | 0.00 |
| R | 0.27 | 0.20 | 0.00 | 0.08 | 0.14 |

Haplogroups have been assigned according to nomenclature in [1].

1. Hammer, M.F., Zegura, S.L. The human Y chromosome haplogroup tree: Nomenclature and phylogeography of its major divisions. Annu. Rev. Anthropol., 2002. 31: p. 303-21.
