# Supplementary material for: The Druze: A Population Genetic Refugium of the Near East
Source: PLoS One. 2008 May 7;3(5):e2105. doi: 10.1371/journal.pone.0002105 (PMC2324201; doi:10.1371/journal.pone.0002105)
Supplement: Figure S1 — (0.28 MB DOC) [file pone.0002105.s007.doc]

**Figure S1 Surname and Household Directed sampling Versus Random sampling**


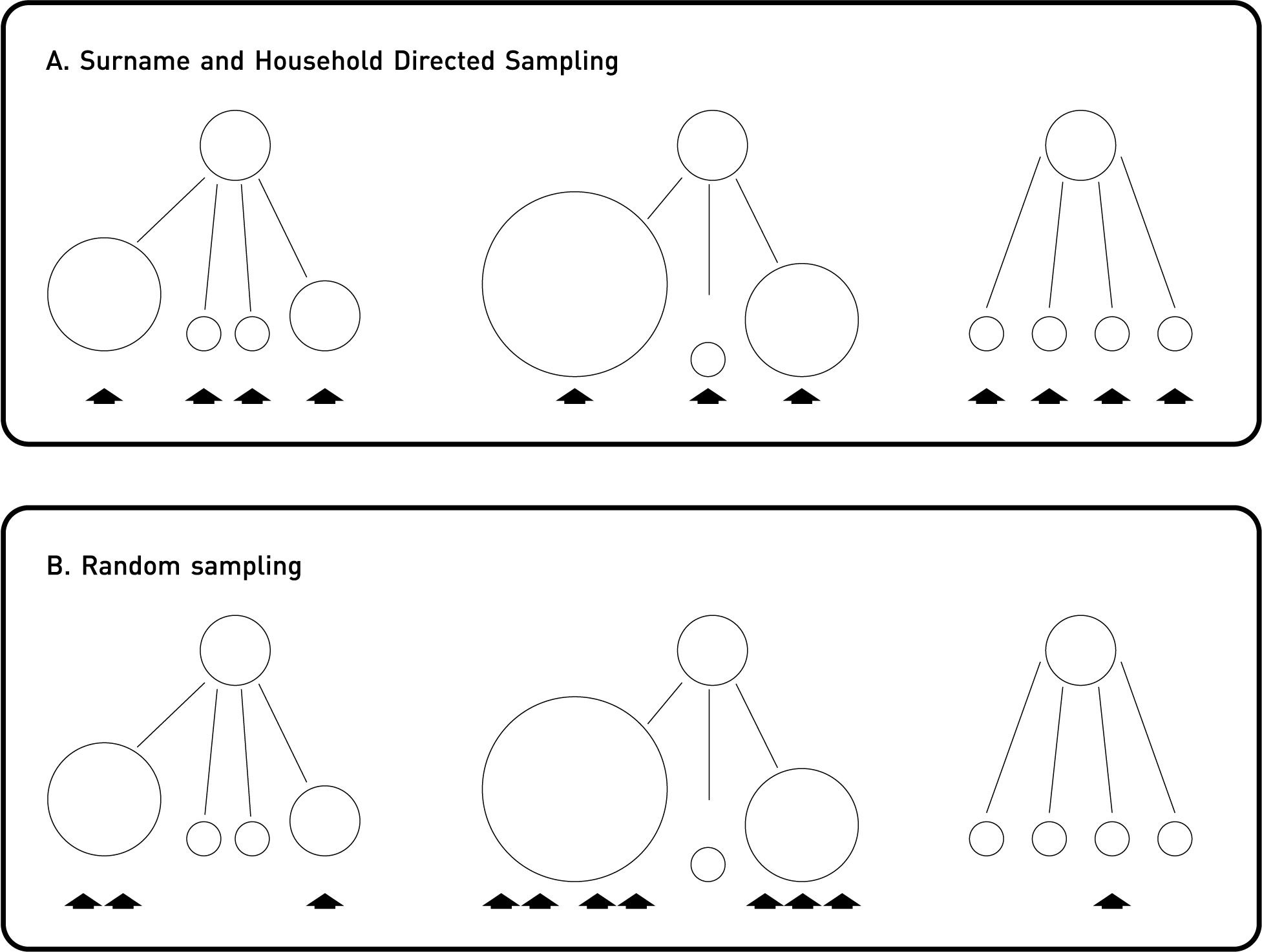


In the current study we have used a surname and household directed sampling instead of the commonly used random sampling.

The terminal nodes represents different households, the size of the terminal node reflects the effective size of the household in the population. The arrows are the sample set in scenario A giving 11 samples from 11 different households (gene diversity H=1 and lower diversity in scenario B (gene diversity H=0.82), reflecting one probable reason for the higher gene diversity in the current study.
