# Supplementary material for: The Druze: A Population Genetic Refugium of the Near East
Source: PLoS One. 2008 May 7;3(5):e2105. doi: 10.1371/journal.pone.0002105 (PMC2324201; doi:10.1371/journal.pone.0002105)
Supplement: Figure S2 — (0.14 MB DOC) [file pone.0002105.s008.doc]

**Figure S2** Migration rates among Druze from different subregions


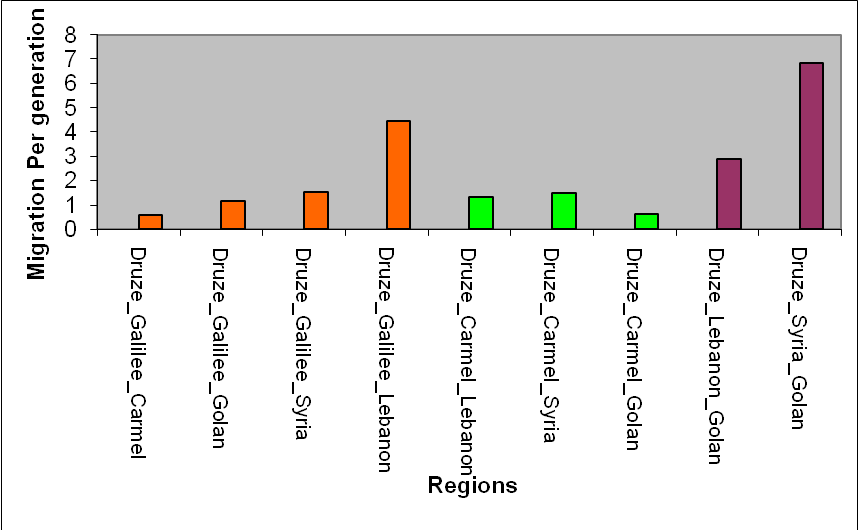


Demographic modeling using the IM application[1] on mtDNA HVS-I sequences (nucleotides 16067-16384) from various Druze subpopulations according to maternal place of birth.

1. Hey, J. and R. Nielsen, Multilocus methods for estimating population sizes, migration rates and divergence time, with applications to the divergence of Drosophila pseudoobscura and D. persimilis. Genetics, 2004. 167(2): p. 747-60.
